# Rapid, High-Capacity, and Reusable Bovine Serum Albumin-Based Adsorbents for Perfluoroalkyl and Polyfluoroalkyl Substance Removal

**DOI:** 10.1021/acsami.5c13467

**Published:** 2026-01-16

**Authors:** Liqing Yan, Elliot Reid, Zefang Chen, Jiahao He, Yongsheng Chen

**Affiliations:** School of Civil and Environmental Engineering, 1372 Georgia Institute of Technology, 200 Bobby Dodd Way, Atlanta, Georgia 30332, United States

**Keywords:** per- and polyfluoroalkyl substances, amine-functionalized bovine serum albumin, water purification, adsorption mechanism, adsorbent

## Abstract

Albumin, a major carrier of perfluoroalkyl and polyfluoroalkyl substances (PFAS) in humans and animals, has been rarely explored for PFAS removal. In this study, bovine serum albumin (BSA) is transformed into an efficient, low-cost, and reusable adsorbent for PFAS removal. BSA is modified with amine-rich poly­(ethylenimine) (PEI) to create a positively charged surface. The optimal PEI:BSA mass ratio was determined to be 0.75 based on a combination of adsorption performance and material properties. The resulting PB(0.75) achieves rapid equilibrium for nine model PFASs, with a pseudo-second order rate constant range from 0.6 to 15.7 g/(mg.min), depending on the specific PFAS. In addition to fast kinetics, PB(0.75) exhibits exceptionally high adsorption capacities for PFHpA, PFOA, PFNA, and PFOS, each exceeding 1000 mg/g. These values rank among the highest reported to date for these PFAS. Adsorption of shorter-chain and emerging PFASs such as PFBA, PFBS, GenX, and 6:2 FTS is comparatively lower but still moderately better than conventional activated carbons reported in the literature. The adsorption mechanism is thoroughly investigated by examining adsorption behaviors under different water matrixes, constructing modified linear solvation energy relationships, and characterizing PB(0.75) before and after adsorption. The adsorption process is governed by electrostatic attraction and hydrophobic interactions, with short-chain PFAS relying more on electrostatic interactions due to their higher hydrophilicity. Furthermore, exhausted PB(0.75) can be effectively desorbed and regenerated by using alkaline solutions. Our study highlights albumin-based adsorbents as a highly promising and sustainable solution for addressing PFAS contamination in aquatic environments.

## Graphical abstract

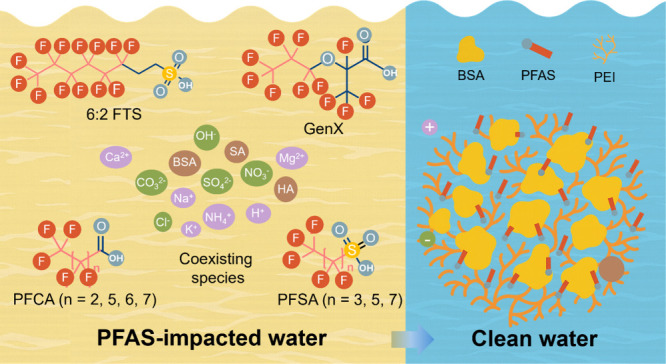

## Introduction

Per- and poly fluoroalkyl substances (PFAS), a class of highly stable fluorinated compounds, have been widely used in industrial applications and consumer products such as nonstick cookware, food packaging, fire- and water-resistant coatings on carpets and clothing, and aqueous film-forming foams (AFFFs).^1^ According to the EPA Toxic Release Inventory (TRI), more than 1.1 million pounds of production-related PFAS waste were reported in 2022, which is likely underreported due to the high reporting threshold and the limited number of PFAS compounds that are required to be disclosed.^2^ Although the production of some legacy PFAS, such as PFOA and PFOS, has been voluntarily phased out in many countries, their persistent nature and the fact that they are the end products of PFAS transformation^3^ mean that they will continue to persist in the environment for years to come. Albumin proteins serve as the major delivery vehicle for PFAS compounds within humans and animals,^4^ which could lead to a series of detrimental effects, such as high occurrence of thyroid disease, cancers, and pregnancy-induced hypertension.^5^ The U.S. EPA has recently established enforceable maximum contaminant levels at 4 ng/L for PFOA and PFOS and 10 ng/L for PFNA, PFHxS, and GenX chemicals.^6^ This presents a significant challenge for remediation efforts, necessitating innovation and sustainable technologies to remove PFAS from water to such low concentrations.

Activated carbon (AC) remains the most technologically mature solution for PFAS control because of its proven effectiveness, established use in water treatment facilities, and comparably low cost.^7^ However, commercially available AC predominately derived from nonrenewable bituminous coals via energy- and chemical-intensive manufacturing processes.^8^ Moreover, AC could be easily saturated during PFAS treatment, especially in the presence of natural organic matter in real-world water, which necessitates either disposal that may be classified as hazardous waste or regeneration that often requires substantial energy inputs. In recent years, various biomass-derived adsorbents such as cellulose fibers, chitosan, proteins,^9^ and cyclodextrins have gained noticeable attention for PFAS removal. Their surfaces are rich in functional groups, including amino, acetamido, and hydroxyl moieties,^10^ which not only enable selective interaction with a broad range of compounds but also facilitate facile surface modifications. Beyond these structural advantages, biomass-derived adsorbents are also renewable, abundant, and biodegradable, making them a more sustainable alternative to conventional AC. While large-scale studies and real-world applications are currently lacking to fully assess the scalability and long-term effectiveness of these adsorbents, preliminary results are undeniably promising. For example, cyclodextrin has consistently outperformed activated carbon in removing short-chain PFAS compounds with fewer than four C–F bonds.^11^

Bovine serum albumin (BSA), an ampholyte protein with both acidic and basic residues, plays a pivotal role as a major delivery vehicle for PFAS within the human body.^4^ Shi et al.^12^ leveraged the affinity between PFAS and BSA to extract 15 legacy PFAS with CF2 > 6 in river water, achieving a recovery rate of 83.8–119.4% for three spiked levels. However, separating BSA after extraction is challenging due to its high solubility in water. Luckily, BSA can be easily converted into insoluble particles through denaturation induced by changes in pH, temperature, reducing agents, or a combination of these factors.^13^ BSA-based particles have demonstrated great removal efficiency for heavy metal ions and dyes,^14,15^ but its application in the removal of PFAS has not been systematically evaluated. Moreover, BSA consists of 538 amino acid residues, which include both carboxyl and amino groups.^16^ These functional groups provide numerous opportunities for surface modification of BSA for specific applications. On the other hand, billions of tons of bovine blood are collected each year but are often used as a low-value animal feed and fertilizer or discarded as waste.^17^ This not only wastes a potentially valuable resource but may also incur additional costs associated with managing the environmental pollution caused by the pathogenicity of bovine blood (e.g., spongiform encephalopathies).^18^ Repurposing bovine blood waste into efficient adsorbents for water treatment is not only a viable and green solution for clean water supply but also significantly benefits waste management.

In this study, BSA was modified with PEI via a facile chemical cross-linking method (Scheme 1) to acquire positive surface charge for enhanced PFAS removal from water. The mass ratio of PEI to BSA was optimized to achieve effective removal of nine model PFAS with varying chain lengths and functional groups. Adsorption kinetic and isothermal investigations were conducted to understand the rate and capacity of PFAS uptake by BSA-based adsorbents under various conditions, including initial PFAS concentration, pH levels, types, and concentrations of natural organic matter and inorganic ions. The spent PB adsorbent was regenerated by using an alkaline washing approach to restore its adsorption capacity. To further elucidate the adsorption sites, the surface chemistry before and after adsorption was carefully characterized and compared. The relationship between the adsorption capacity and the interactions between PB and ionizable PFAS was successfully described by a modified linear solvation energy relationship (LSER). These outcomes will facilitate the valorization of protein waste into an efficient PFAS adsorbent, addressing the critical issue of PFAS contamination and reducing food waste.

**Scheme 1:**
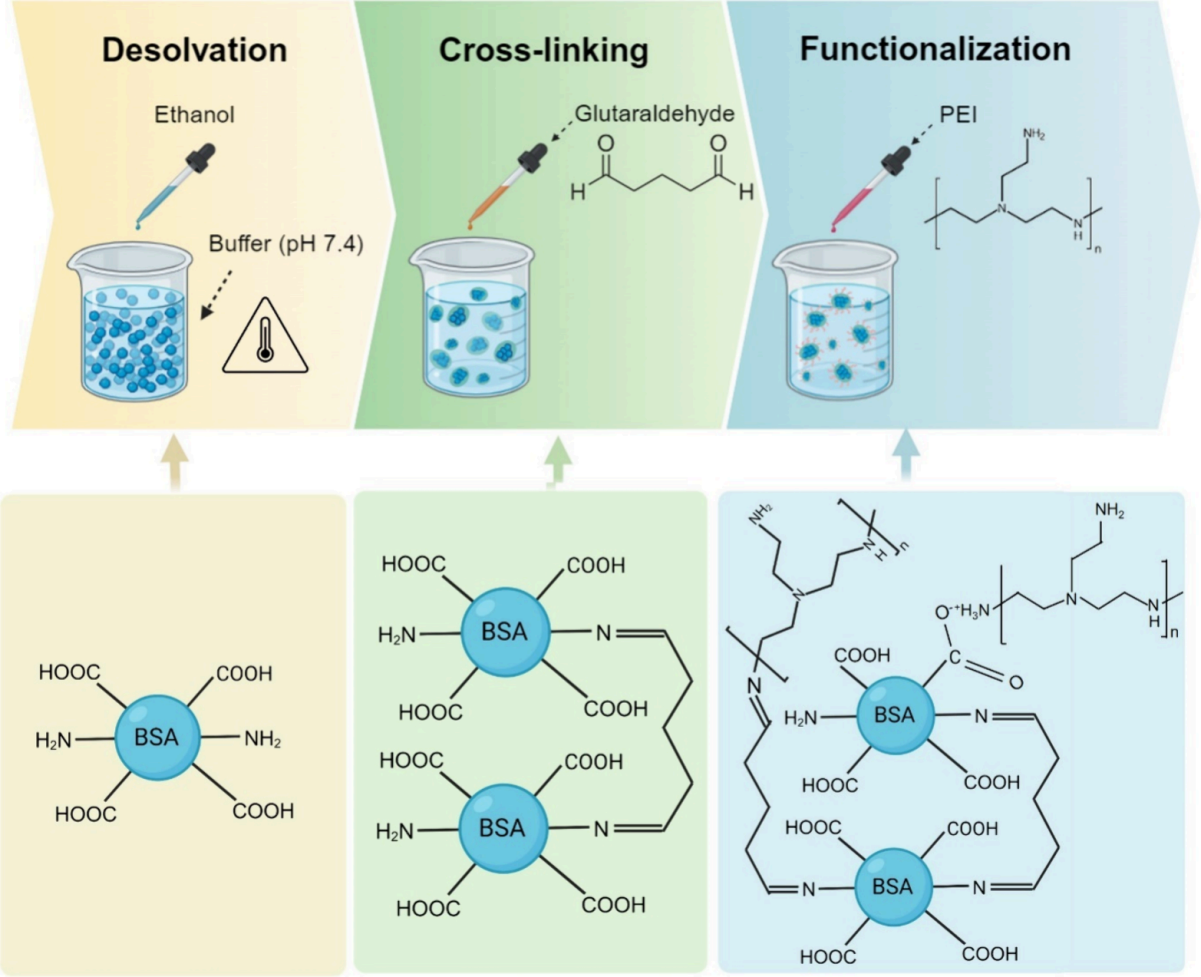
Synthesis of PEI-Functionalized BSA Nanoclusters (PBs)

## Materials and Methods

### Chemicals

BSA (Mw: ∼66 kDa, ≥ 98%), branched PEI (average Mw ∼800), and glutaraldehyde were purchased from Sigma-Aldrich and used without further purification. PFAS studied in this work involved perfluorocarboxylic acids (PFCAs, C4, C7, C8, and C9) and perfluorosulfonic acids (PFSAs, C4, C6, and C8) were also purchased from Sigma-Aldrich, while GenX and 6:2 FTS were obtained from Toronto research chemicals. Phosphate buffer solution (PBS, 10 mM, pH = 7.4) was prepared by mixing Na_2_HPO_4_ and NaH_2_PO_4_ stock solution. Ultrapure water with a resistivity of 18.2 MΩ cm^–1^ was used to prepare all of the aqueous solutions.

### Synthesize PEI-Functionalized BSA Nanoclusters

A 1 g portion of BSA powder was dissolved in 40 mL of 10 mM phosphate buffer (pH 7.4) to get a clear solution. After adding 60 mL of ethanol, the solution (1 wt % of BSA) was incubated at 65 °C for 24 h and then at room temperature for 2 days to get the BSA nanocluster suspension, which was freeze-dried and named BNP. A 100 mg portion of BNP was resuspended into 40 mL of 10 mM PBS buffer. Four milliliters of 10% glutaraldehyde was added to the suspension and stirred at 200 rpm for 2 h at 4 °C. Then, the solution was centrifuged and resuspended in 40 mL of PBS solution. A certain amount of 10% PEI aqueous solution was then added to the resuspended albumin solution, and the mixture was stirred at 200 rpm for 1 h at room temperature. Then, the solids were collected by centrifugation and were washed three times with PBS to remove excess PEI and GTH. The prepared materials were named PB­(*x*), where *x* indicates the mass ratio between PEI and BNP, *x* = 0, 0.25, 0.5, 0.75, and 1.0.

### Measurements and Characterizations

The FTIR spectra of PBs before and after adsorption were recorded on a Shimadzu IRAffinity FTIR spectrometer with a scanning wavenumber of 400–4000 cm^–1^. XPS analysis was conducted on a Thermo K-Alpha X-ray photoelectron spectrometer. The zeta potentials were measured with a Malvern zetasizer. Two milligrams of PBs was suspended in 10 mL of 0.01 M NaCl solution for the measurement. Thermo Fisher Helios 5CX was used to take SEM and EDX images of PB(0.75) at −140 °C.

LC-MS-MS was used to quantify the target PFAS in the prepared samples. An InfinityLab Poroshell 120 EC-C18, (2.7 μm, 2.1 mm × 150 mm) was used with a working temperature of 50 °C. Aqueous ammonium acetate (5 mM) solution and methanol/acetonitrile (80:20%, v/v) were chosen as binary mobile phase solvents A and B, respectively, and their flow rates were both fixed at 0.25 mL/min. The mobile phase gradient is shown in Table S5. Calibration curves were calculated at the beginning of the analytical sequence. Instrument blanks were run before and after the calibration curve. For high-concentration PFOA measurement used during the regeneration experiments, an Agilent HPLC is used. Na_2_HPO_4_ (0.25 mM, adjust pH = 2 with phosphoric acid) and acetonitrile (50%/50%, v/v) were used as the mobile phase. An Agilent Eclipse Plus C18 column (3.5 μm, 4.6 mm × 150 mm) was used as the analytical column.

### Batch Adsorption Experiments

The kinetics experiments were performed in 250 mL polypropylene (PP) flasks. Specifically, 100 mL of ultrapure water was added into the tube, and then, 100 μL of PFAS stock solution (100 mg/L) was spiked to maintain an initial concentration of 100 μg/L for individual PFAS. The pH of the solution was adjusted to 7 with HCl or NaOH (0.1 mol/L). After vertexing, 1 mL of the PFAS solution was withdrawn and used for measurement for 0 min. Once 20 mg of PBs was added into the solution, the flasks were shaken at room temperature and aqueous samples were withdrawn at 5, 10, 15, 20, 25, 30, 40, 50, and 60 min. The collected samples were then centrifuged at 14,000 rpm for 2 min as a satisfactory recovery rate was not achieved with PES syringe filters, especially for PFOS (Figure S19). By contrast, mass loss during centrifugation was only observed when the concentration becomes extremely high (e.g., 2000 mg/L PFNA), which is far beyond our experimental range (Figure S20). The supernatant was kept refrigerated until further measurements. Experiments were performed in triplicate, and the results were reported as an average ± standard deviation. The collected data were fitted to pseudo-first-order eq 1 and pseudo-second-order eq 2 models.

$$\mathrm{pseudo}-\mathrm{first}-\mathrm{order}:\;\ln=\ln q_{e}-k_{1}t$$ 1

$$\mathrm{pseudo}-\mathrm{second}-\mathrm{order}:\frac{t}{q_{t}}=\frac{1}{k_{2}q_{e}^{2}}+\frac{t}{q_{e}}$$ 2

where *q*_t_ and *q*_e_ (mg/g) are the amount of adsorbate in the solid phase at time *t* (min) and at equilibrium, respectively, and *k*_1_ (min^–1^) and *k*_2_ (g/(mg·min)) are the rate constants.

To determine the maximum adsorption capacity of PB, isotherm experiments were conducted in 50 mL PP centrifuge tubes containing 40 mL of PFAS solution at varying concentrations, with the PB dosage fixed at 20 mg/L. The solution pH was adjusted to 7 using 0.1 mol/L HCl or NaOH. The tubes were shaken at room temperature for 2 h to allow adsorption equilibrium, after which samples were withdrawn and centrifuged for analysis. Adsorption data were fitted to two traditional isotherm models: Langmuir and Freundlich, given by eqs 3 and 4, respectively.

$$\mathrm{Langmuir}:\;q_{e}=\frac{q_{m}K_{L}C_{e}}{1+K_{L}C_{e}}$$ 3

$$\mathrm{Freundlich}:\;q=K_{F}C_{e}^{1/n}$$ 4

where *q*_m_ (mg/g) is the theoretical adsorption capacity, *C*_e_ (mg/L) is the concentration of adsorbate in the aqueous phase at equilibrium, *K*_L_ (L/mg) is the Langmuir constant, *K*_F_ (mg/g)/(mg/L)^1/*n*^ is the Freundlich constant related to adsorption capacity and energy, and *n* is the dimensionless heterogeneity coefficient indicating the favorability of the adsorption process.

To evaluate the effects of water matrixes, adsorption experiments were carried out in 100 mL polypropylene bottles containing 10 mg/L PB(0.75) and 100 μg/L PFAS at pH 7 and room temperature unless otherwise specified. To examine the effect of the initial PFAS concentration, solutions of 100, 500, 1000, and 5000 μg/L were tested. The influence of pH was assessed by adjusting solutions to pH 4, 7, or 10 with 0.1 M NaOH or HCl. The effect of natural organic matter (NOM) was evaluated by adding humic acid at 0.1, 1, 5, and 10 mg/L, along with 5 mg/L bovine serum albumin (BSA) and sodium alginate (SA) as model NOM components. To assess the role of coexisting ions, 0.5 mM MgCl_2_, CaCl_2_, Na_2_SO_4_, and Na_2_CO_3_, as well as 1 mM KCl, NH_4_Cl, NaNO_3_, and NaCl, were introduced. Samples were collected at 0, 5, 10, 15, 20, 25, 30, 40, 50, and 60 min, then centrifuged, and refrigerated prior to analysis.

### LSER Model

LSER model is used to explain the partition of PFAS between water and PB. For nonionic solutes, Abraham and co-workers^200^ identified the intermolecular interactions as dispersion, cavity formation, dipolar interactions, and hydrogen-bonding interactions and formulated a group of parameters (solute descriptors) to describe and predict these interactions.^51^

logK=c+eE+sS+aA+bB+vV 5

where log *K* is the partitioning coefficient of the adsorbate between the absorbent and water under equilibrium conditions. *E* is the excess molar refraction in (cm^3^/mol)/10 that represents nonspecific van der Waals forces. *S* is the polarizability/dipolarity parameter, measuring the dipolar interactions. *A* and *B* have hydrogen-bond donating (acidity) and accepting abilities (basicity), respectively. *V* is the molecular volume or McGowan’s volume in (cm^3^/mol)/100, which is linked with the hydrophobically driven adsorption as well as nonspecific interactions between adsorbate and adsorbent. *c* is the regression constant, while *e*, *s*, *a*, *b*, and *v* are the fitting coefficients for each interaction, indicating the relative contribution of each descriptor to the adsorption process. Positive and negative signs of the coefficient suggest positive and negative impacts, respectively.

Equation 5 is often used for partitioning between two condensed phases. Goss^52^ proposed eq 6 as a universal model to describe both condensed–condensed and gas-condensed phase partitioning and was found to work better for polyfluorinated compounds because the inclusion of *E* sometimes led to a substantial error.^53^

logK=c+sS+aA+bB+lL+vV 6

*L* is the logarithmic hexadecane-air partition coefficient that is correlated with the strength of the nonspecific van der Waals interactions.^52^ *l* is the fitting coefficient for *L*. Other parameters have the same meaning as in eq 5.

Both eqs 5 and 6 were developed for nonionic molecules, and an additional descriptor *P* (scaled effective acid dissociation constant)^54^ is introduced in this study to account for the ionization status of PFAS molecules. The modified equations are eqs 7 and 8.

logK=c+eE+sS+aA+bB+vV+pP 7

logK=c+sS+aA+bB+lL+vV+pP 8

where *P* is the scaled effective acid dissociation constant, calculated as (14 – p*K*_a_)/10. *p* is the fitting coefficient for *P*.

Experimentally measured descriptors are generally not available for PFAS. Solvatochromic descriptors for PFAS were obtained from the Helmholtz Centre for Environmental Research^55^ and are listed in Table S6. Compounds’ SMILES files were inserted into the computational tool to calculate the descriptors. p*K*_a_ values of PFAS, which are required to calculate the *D* and *P* values, were estimated and obtained from EPA’s CompTox database. Multiple linear regression with a stepwise approach was employed to develop correlations between independent descriptors and absorbability toward PFAS. Fitting equations were obtained by using SPSS software. The goodness of model fit was examined by coefficient of determination (adjusted *R* ^2^). The regression models were evaluated by the *p*-values presented in the analysis of variance (ANOVA), with a *p*-value less than 0.05 indicating that at least one of the independent descriptors of the developed equations is useful in predicting the dependent variable at a 95% level of significance. The predictive precision of the models was checked by the root-mean-square error (RMSE), which is calculated by dividing the squared differences between the predicted and actual values by the number of PFAS involved and then taking a square root of the result.

### Regeneration

A 0.1 M NaOH solution was used to regenerate exhausted PB(0.75). Typically, 0.8 mg of PB(0.75) was placed in contact with 1.5 mL of a 200 mg/L PFOA solution for 2 h to reach equilibrium. The adsorbent was then separated via centrifugation, and 1.5 mL of 0.1 M NaOH was added and shaken for 10 min. After another round of centrifugation, PB(0.75) was washed extensively with Milli-Q water to neutralize the solution. Finally, the adsorbent was dried at 65 °C overnight, making it ready for use in the next cycle.

## Results and Discussion

### Adsorbent Synthesis and Characterization

PEI-functionalized BSA nanoclusters (PB) were prepared via a cross-linking approach (Scheme 1). Gradually adding water-soluble ethanol to the BSA solution reduced the hydration level and solubility of BSA in water, leading to saturation and the formation of protein nuclei. Free protein units then condense around these nuclei, growing into nanoclusters.^19^ Glutaraldehyde was subsequently added as a cross-linker to stabilize the nascent nanoclusters. The aldehyde (R–CHO) of GTH could react with primary amines on BSA, forming Schiff bases (R–C=NH) with the concomitant release of water.^20^ As a bifunctional reagent, GTH could bridge two amino groups, either from separate BSA molecules or from different regions of the same molecule, depending on spatial orientation, thereby establishing an extensive cross-linked network. Finally, branched PEI was added to functionalize the clusters with additional amine groups. PEI can be grafted onto the surface of BSA nanoclusters not only through GTH-mediated cross-linking but also via electrostatic interactions arising from its positive charge.^21^

A key parameter during the adsorbent synthesis is the ratio of BSA, PEI, and GTH. To determine the optimal mass ratio between GTH and BSA, nine PFAS including PFCA (PFBA (C4), PFHpA (C7), PFOA (C8), PFNA (C9)), PFSA (PFBS (C4), PFHxS (C6), and PFOS (C8)) and replacement, PFAS (GenX (C6) and 6:2 FTS (C8)), were chosen as model PFAS in this study, whose structures are provided in Table S1. When the PEI:BSA mass ratio is fixed at 0.75, increasing the GTH:BSA mass ratio from 2 to 4 leads to improved removal efficiencies for short-chain PFAS: PFBS removal at 5 min increases from 75% to 88%, PFBA increased from 7% to 28%, and GenX increases from 58% to 73% (Figure S1). PFHxS, PFOS, PFHpA, PFOA, PFNA, and 6:2 FTS show no comparable improvement, as their removal is already nearly complete, even at GTH:BSA = 2. Further increasing the GTH:BSA ratio beyond 4 does not yield additional gains in PFAS uptake, presumably because the excess cross-linker cannot engage new functional groups. As the observed changes with varying GTH:BSA ratios were relatively minor and reached a plateau quickly, we did not explore this parameter in greater depth and adopted GTH:BSA = 4 for the sake of the study. In contrast, we systematically varied the PEI:BSA ratio (0, 0.25, 0.5, 0.75, and 1.0), which exhibit more pronounced differences, and compared their structural characteristics and PFAS adsorption performance in greater detail below.

XPS was used to evaluate the surface chemistry of BSA before and after functionalization with PEI. According to the XPS survey scan (Figure 1a), PBs are made of C, N, and O and a trace amount of S. With the grafting of PEI, the most noticeable change in the elemental composition is the decrease in the concentration of O and the increase in the concentration of C. The grafting of PEI introduces additional C and N in the material, which may make the relative percentage of O decrease. The high-resolution XPS spectra of PB(0.75) were recorded to provide more detailed information on its chemical status. Three peaks at 284.6, 285.8, and 287.5 eV in C 1s spectra can be attributed to C-1 (C–C, C=C, and C–H), C-2 (C–NH), and C-3 (C–O), respectively (Figure 1b). N-1 (C–N) and N-2 (NH_2_) are positioned at 399.1 and 400.7 eV, respectively (Figure 1c). The O-1 (C=O) and O-2 (O–H) are located at about 531, and 532 eV, respectively (Figure 1d). These peak assignments are consistent with previous studies on BSA nanoparticles.^22^

**Figure 1:**
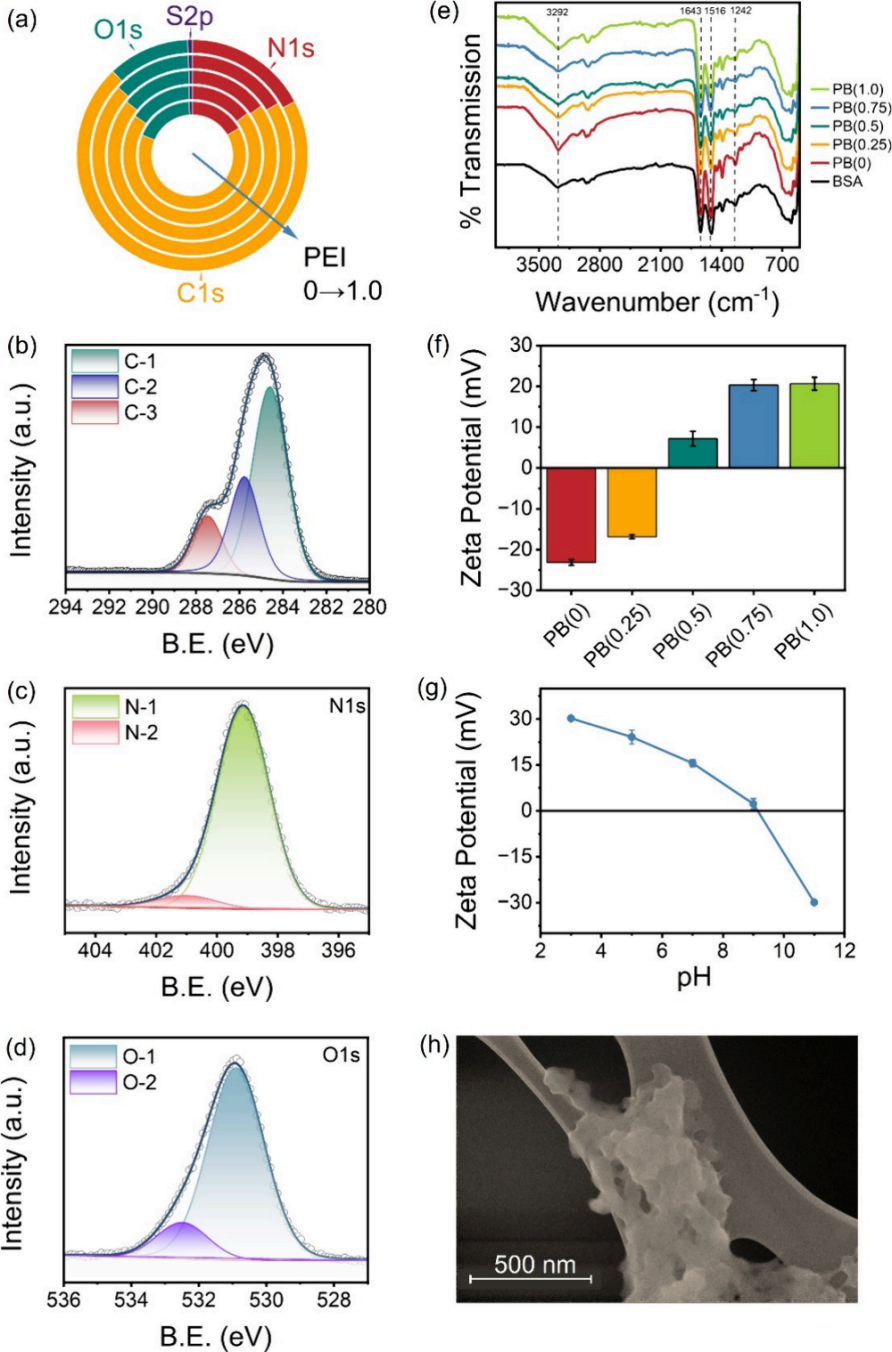
Characterizations of PBs. (a) XPS survey results of PB­(x), where *x* = 0. 0.25, 0.5, 0.75, and 1.0. (b) C 1s, (c) N 1s, and (d) O 1s high-resolution XPS spectra for PB(0.75). (e) FTIR spectra of BSA and PB­(*x*). (f) Zeta potential of PB­(*x*) materials at pH 7. (g) Zeta potential versus pH relationship of PB(0.75). (h) SEM image of PB(0.75).

The functional groups of BSA and PBs were evaluated by FTIR spectroscopy (Figure 1e). In pristine BSA, the broad peak at 3292 cm^–1^ was associated with O–H or N–H stretching vibrations, which is attributed to the presence of amine groups on BSA. The strong vibration peaks at 1643 cm^–1^ and 1516 cm^–1^ correspond to the stretching vibrational peaks of the amide I band (C=O) and the amide II band (in-plane NH bending), respectively, while 1242 cm^–1^ is the vibrational peak of the amide III band (C–N).^23^ Upon PEI functionalization (PB­(*x*)), these characteristic peaks were retained, suggesting the preservation of the protein backbone. Discrete new peaks are not expected because PEI is rich in primary/secondary/tertiary amines whose characteristic bands are also N–H stretch near 3300 cm^–1^, N–H bending around 1600 cm^–1^, and broad C–N stretching across 1350–1000 cm^–1^, overlapping those of BSA.^24^ Accordingly, only subtle peak position shifts attributable to altered hydrogen bonding and local microenvironments are observed (Figure S2). Finally, the spectra lack aldehyde markers (i.e., a strong C=O band at 1740–1720 cm^–1^), suggesting minimal residual glutaraldehyde after grafting.

BSA has both carboxyl and amine groups, which allow it to demonstrate pH-dependent surface charges. Figure 1f illustrates the zeta potential of PBs at pH 7 with an ionic strength of 100 mM. As the PEI loading rate increases, the zeta potential of PBs also increases. This increase is likely due to a higher proportion of protonated amine groups and a lower proportion of deprotonated carboxyl groups, resulting from the grafting of more PEI. Since most PFAS are negatively charged in environmentally relevant pH,^25^ cationic PBs are promising to interact with PFAS via electrostatic attraction. The relationship between solution pH and zeta potential was further evaluated with PB(0.75) (Figure 1g). At acidic conditions, the carboxyl groups and amine groups protonated to COOH and NH_4_^+^, respectively, resulting in a positive zeta potential due to the dominance of the protonated amine groups. As the pH increases, the carboxyl groups start to deprotonate to form COO^–^, while the amine groups remain protonated. This can lead to a zeta potential close to zero. PB(0.75) has a point of zero charge around pH 9.1, significantly higher than BSA’s isoelectric point, which is in a range of pH 4.5–5.0.^26,27^ With a further increase in pH, the carboxyl groups are fully deprotonated (COO^–^), and the amine groups start to deprotonate to form NH_3_. This results in a negative zeta potential due to the dominance of the deprotonated carboxyl groups.

Scanning electron microscopy (SEM) was used to examine the surface morphology of PB(0.75). At a suspension concentration of 0.5 mg/mL, PB(0.75) appears as elongated fibrils with lengths of ∼100 nm and widths of ∼30 nm (Figure S3). Increasing the concentration to 5 mg mL^–1^—comparable to the adsorbent dosage used in PFAS uptake experiments—yields larger, clustered assemblies (Figure 1h). This concentration-dependent transition might be driven by the high surface energy of small particles and elevated collision frequency at higher adsorbent dosages. In addition, the GTH used during synthesis can bridge PEI and BSA, promoting formation of an extended network (Scheme 1).

### Optimize PEI Loading Rate for PFAS Removal

The optimal PEI:BSA ratio for PFAS adsorption was first evaluated. As shown in Figure 2, BSA nanoclusters without PEI functionalization (PB(0)) exhibited negligible PFAS removal, likely due to their negative surface charge (zeta potential = −23.1 mV at pH 7), which causes electrostatic repulsion and hinders adsorption. Upon PEI functionalization, the removal efficiency increased progressively. Complete removal was achieved for PFOA, PFNA, PFHxS, PFOS, and 6:2 FTS, while 80–90% removal was obtained for PFHpA, PFBS, and GenX once the PEI:BSA mass ratio exceeded 0.5. The improvement is attributed to the amine groups introduced by PEI, which increase the surface positive charge, as confirmed by zeta potential measurements (PB(0.25): −16.9 mV; PB(0.5): +7.2 mV at pH 7). Further increasing the ratio from 0.5 to 0.75 enhanced the positive charge, although adsorption performance showed little additional gain under the tested conditions, likely because PB(0.5) already achieved near-complete removal for many PFAS. At a PEI:BSA ratio of 1, no additional improvement was observed in adsorption performance, and the properties (e.g., zeta potential) were nearly identical to those of PB(0.75). This is most likely because the available active sites on BSA had already been saturated and could not accommodate additional PEI binding. Thus, PB(0.75) was identified as the most promising and cost-effective formulation for further investigation.

**Figure 2:**
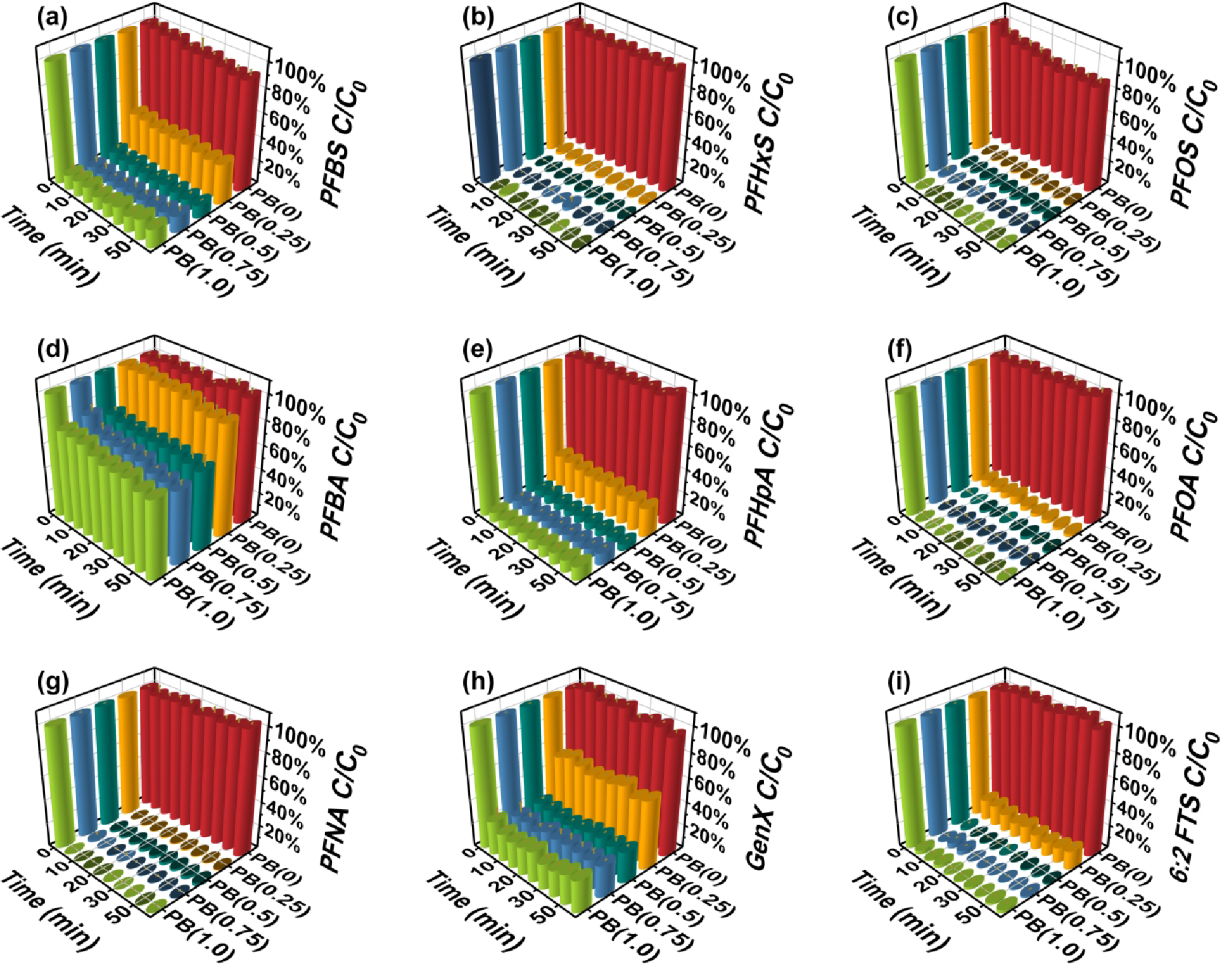
Concentration versus time relationship for (a) PFBS, (b) PFHxS, (c) PFOS, (d) PFBA, (e) PFHpA, (f) PFOA, (g) PFNA, (h) GenX, and (i) 6:2 FTS using PB­(*x*), *x* = 0, 0.25, 0.5, 0.75, and 1. Other experimental conditions were: initial concentration of PFAS: 100 μg/L, adsorbent concentration: 200 mg/L, pH = 7, and temperature = 25 °C.

It is also noticeable that the adsorption reaches equilibrium within 5 min for both short- and long-chain PFAS under studied conditions (initial concentration of PFAS: 100 μg/L, adsorbent concentration: 200 mg/L, pH = 7, and temperature = 25 °C), indicating that the adsorption sites on PBs are highly accessible and that the driving forces for adsorption are strong. Adsorption data on PB(0.75) were fitted with pseudo-first-order (PFO) and pseudo-second-order (PSO) models, and the results are shown in Table S2. Although both models provided good fits, PSO showed a higher *R* ^2^ values for all PFAS and the *q*_e_ values calculated were closer to the measured ones. This suggests that the PSO models were able to describe the adsorption kinetics of all of the studied PFAS well. While PSO is often associated with chemisorption,^28^ in this case, the good fit more likely reflects a stronger rate dependence on the remaining adsorption capacity. The fitted *k*_2_ values range from 0.554 to 11.654 g/(mg·min), markedly faster than those reported for conventional activated carbons. For example, reported *k*_2_ values for PFOA and PFOS on various activated carbon fall within 0.001–2.050 g/(mg·min) and 0.010–0.905 g/(mg·min), respectively.^29^ In contrast, PB(0.75) achieved *k*_2_ values of 11.195 g/(mg·min) for PFOA and 2.148 g/(mg·min) for PFOS. Considering that most water treatment plants operate with adsorption column contact times less than 60 min (some even less than 20 min),^30^ the exceptionally rapid adsorption kinetics of PB(0.75) represent a significant practical advantage.

### Isotherm Studies

To quantitatively analyze the maximum adsorption capacity of PB(0.75) against PFAS, isotherm studies were conducted and the results are shown in Figure 3. Langmuir and Freundlich models were used to fit the isotherm data, and the fitting parameters are summarized in Table 1. Langmuir and Freundlich both provided good fitting for PFHpA, PFOA, PFNA, PFHxS, and PFOS, while only Freundlich was appropriate for PFBA, PFBS, GenX, and 6:2 FTS. This suggests that the Freundlich model is more suitable to describe the adsorption of PFAS on PB(0.75). The result indicates that the adsorption process takes place on heterogeneous (multilayer) surfaces, which aligns with the heterogeneous morphology of PB(0.75) revealed by the SEM image. When compared the adsorption capacities with other adsorbents in the literature (Table S3), PB(0.75) stands out as one of the best, especially for long-chain PFCAs and PFSAs. Notably, the adsorption capacity of PB(0.75) exceeds 1000 mg/g for PFHpA, PFOA, PFNA, and PFOS, which is among the highest reported.^7^

**Table 1:** Isotherm Fitting Results and the Measured Maximum *q*_e_

	$q_{e}=\frac{q_{m}K_{L}C_{e}}{\left(1+K_{L}C_{e}\right)}$			$q_{e}=K_{F}C_{e}^{1/n}$			
PFAS	*q*_m_(mg/g)	*K*_L_(L/mg)	*R* ^2^	*K*_F_ (mg/g)/(mg/L)^1/*n*^	*n*	*R* ^2^	*q*_max_measured_(mg/g)
PFBA	9.3	0.35	0.0257	3.10	1.01	0.9946	0.30
PFHpA	1215.3	0.05	0.9491	78.61	1.67	0.9793	886.29
PFOA	1111.1	0.15	0.9938	197.20	2.48	0.9778	1040.85
PFNA	1329.7	0.18	0.9893	216.77	2.19	0.9598	1303.25
PFBS	3.0	3.58	0.6193	5.28	1.20	0.9901	1.12
PFHxS	454.5	0.13	0.9884	77.27	2.43	0.9834	417.70
PFOS	1213.0	0.71	0.9729	230.71	2.10	0.9140	1261.77
GenX	1.8	3.66	0.6597	3.65	1.15	0.9921	0.60
6:2 FTS	4.0	11.22	0.7827	25.95	1.09	0.9903	1.26

**Figure 3:**
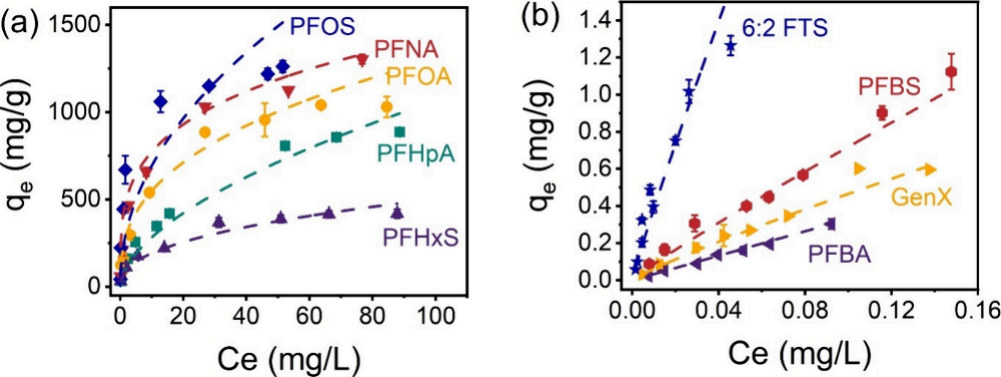
Isotherms data for (a) PFOS, PFNA, PFOA, PFHpA, and PFHxS and (b) 6:2 FTS, PFBS, GenX, and PFBA on PB(0.75). The symbols with error bars are experimental data points, while the dashed lines are the Freundlich fitting of the corresponding PFAS.

The adsorption capacities vary greatly and follow the order of PFNA ≈ PFOS > PFOA > PFHpA > PFHxS > 6:2 FTS > PFBS > GenX > PFBA. The Langmuir maximum adsorption capacities are reasonably close to the maximum uptake experimentally measured, which confirms that the reported adsorption capacities are physically reasonable and not artifacts of data processing. Clearly, the adsorption capacity positively correlates with the carbon chain length of PFAS. As the carbon chain length increases, so does the hydrophobicity, thereby enhancing the likelihood of hydrophobic interactions with PB(0.75). Moreover, longer fluorocarbon chains provide a greater nonpolar surface area for stronger dispersive interactions with the surface of PB(0.75).^31^ On the other hand, the removal efficiency of sulfonated PFAS (PFSA) was higher than carboxylic PFAS (PFCA) with the same chain length. The sulfur–oxygen bond in a sulfonic acid group has a higher negative charge density than the carbon–oxygen bond in a carboxylic acid, and the molecular volume of the sulfonate functional group is larger than that of the carboxylate functional group.^32^ These attributes make both electrostatic and hydrophobic interactions more favorable for PFSAs. Similar trends were observed for the adsorption of PFAS on other adsorbents such as activated carbons,^7^ ion exchange resins,^33^ and the metal–organic framework.^34^

### Adsorption Mechanism Investigation

#### Influences of Initial PFAS Concentration, pH, and Coexisting Species

The actual adsorption performance is governed by a variety of factors, including the initial concentration of PFAS, pH levels, dissolved organic matter, and the number of inorganic ions. These components can interact with both PFAS and PB(0.75) in complex ways to enhance or hinder the adsorption process. Probing these interactions is essential for optimizing the removal of PFAS from water by PB(0.75) and elucidating the underlying interaction mechanisms.

PFAS are found in contaminated water sources at concentrations ranging from low ng/L in surface water to hundreds of mg/L in AFFF.^35–37^ Therefore, initial concentration ranges from 100 to 5000 μg/L were selected to study the influence of PFAS concentration on removal efficiency. Figure 4 shows the removal rate of PFAS at various initial concentrations after 1 h of contact with PB(0.75), while Figure S4 provides the detailed time–concentration relationship. When the initial concentration of PFAS was below 1 mg/L, the removal rate slightly decreased as the initial concentration increased. This phenomenon can be attributed to the abundant availability of adsorption sites on PB(0.75), which are easily accessible at lower concentrations. Short-chain PFAS were more sensitive to the increase in the initial concentration than the longer ones. For example, the removal efficiency of PFBS and PFOS was reduced by 75% and 11% when the initial concentration increased from 100 to 1000 μg/L. This might be because fewer adsorption sites are available for short-chain PFAS, as they rely more heavily on electrostatic attraction while both electrostatic attraction and hydrophobic interactions contribute to the adsorption of long-chain PFAS. When the initial concentration of PFAS further increased to 10 mg/L, a significant drop in adsorption efficiency was observed for all PFAS, which might be due to the saturation of the available adsorption sites.

**Figure 4:**
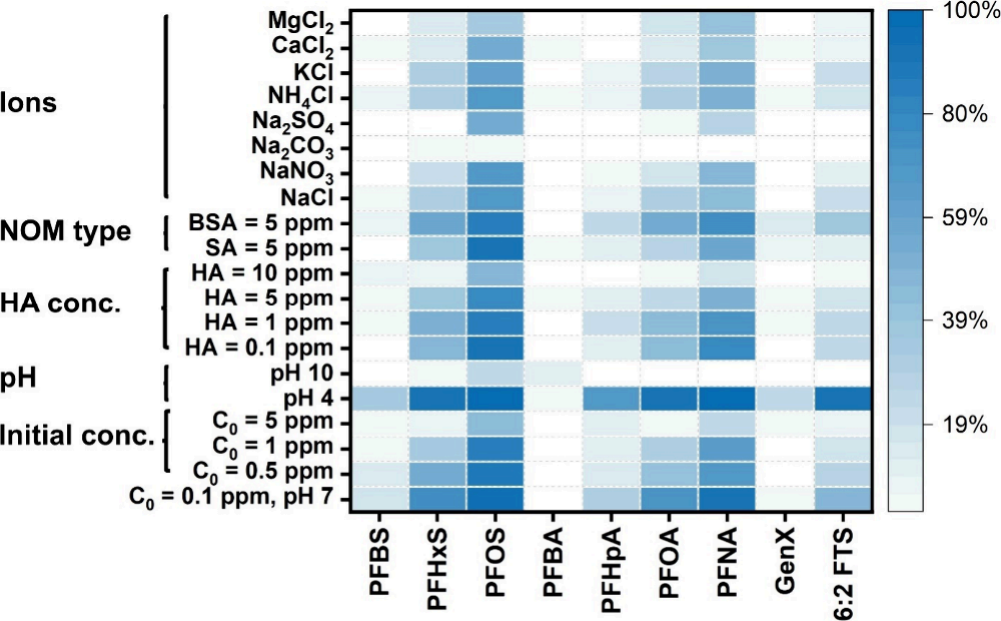
Influences of the matrix on the adsorption of PFAS on PB(0.75).

PB­(0.75) exhibits pH-dependent surface charges with an isoelectric point around 9.1, which likely leads to a pH-dependent adsorption performance. Meanwhile, most PFAS exist in the anionic form across a wide range of pH values due to their low p*K*_a_ values.^38,39^ Consequently, the solution pH may affect the adsorption of PFAS by changing both the adsorbent surface charge and the adsorbate species. As shown in Figure 4 and Figure S5, pH has a critical effect on the removal efficiency of PFAS by PB(0.75). At acidic conditions, the functional groups on the PB(0.75) surface would be protonated, leading to enhanced electrostatic attractions between the positively charged PB(0.75) surface and anionic PFAS compounds in the aqueous solution. Consequently, the removal rate increased significantly for most PFAS when the pH was lower than 4, especially for short-chain ones. For example, the removal rate for PFBS increased by 87% at pH 4 when compared to pH 7, meanwhile, the removal rate of PFHxS only increased by 23%. This difference may be attributed to the more significant role of electrostatic interactions in short-chain PFAS adsorption. However, it should be noted that the minimal increase in the PFOS and PFNA removal rate does not necessarily mean pH does not influence the adsorption of these PFAS. Near-complete removal of these PFAS was already achieved at pH 7, which is advantageous for practical applications in the real world. When the pH was increased to 10, negligible adsorption was observed for all PFAS except for PFOS, but the removal rate of PFOS was also greatly reduced by 74%. The negative charge on PB(0.75) at pH 10 would cause electrostatic repulsion with PFAS, suggesting that electrostatic attraction is essential for the adsorption of all PFAS onto PB(0.75), regardless of their hydrophobicity.

Coexisting components, which are omnipresent in natural waters, have been reported to pose great challenges in the practical application of adsorption for PFAS removal. The composition and concentration of these components vary greatly depending on the water source (e.g., groundwater, rivers, lakes, and streams) as well as surrounding land use, climatic conditions, geology, and other environmental factors. For example, groundwater typically contains relatively low levels of dissolved organic carbon (DOC) but higher total dissolved solids (TDS), whereas surface waters often exhibit higher DOC but lower TDS. Beyond magnitude, the chemical makeup of DOC and TDS also shifts across settings. To reflect this variety in water chemistry, we evaluated PFAS removal by PB(0.75) using representative natural organic matter and salts across realistic concentrations: humic acid (0.1, 1, 5, and 10 mg L^–1^), bovine serum albumin (BSA, 5 mg L^–1^), and sodium alginate (SA, 5 mg L^–1^) to represent humic substances, proteins, and polysaccharides, respectively, and 1 mequiv L^–1^ of NaCl, NaNO_3_, Na_2_CO_3_, Na_2_SO_4_, NH_4_Cl, KCl, MgCl_2_, and CaCl_2_ to probe inorganic effects. This design enables a controlled yet representative assessment of PB(0.75) under conditions approximating those of real-world waters.

HAs at concentrations ranging from 0.1 to 10 (1 to 100 times the concentration of PFAS) were first added to the solution to investigate their effects on the adsorptive removal of PFAS by PB(0.75). As the concentration of HA increases, its inhibitory effects on the adsorption of PFAS become more pronounced (Figure 4 and Figure S6). When the HA concentration reached 10 ppm, adsorption was inhibited for most PFAS, except for PFOS, which showed a 50% reduction in the removal rate compared to DI water. This may be due to the negative charge carried by HA that allows it to compete with PFAS for adsorption sites on the surface of PB(0.75). As a result, the higher concentration of HA reduces the availability of adsorption sites for PFAS, thereby decreasing the efficiency of PFAS removal. On the other hand, short-chain PFAS are more susceptible to the addition of HA. For instance, when the HA concentration was 5 ppm, the removal rates of PFBS, PFHxS, and PFOS decreased by 88%, 50%, and 18% compared to DI water, respectively. This might be due to the greater significance of electrostatic attraction in the adsorption of short-chain PFAS. The impact of different types of natural organic matter was also investigated using HA, BSA, and SA. As shown in Figure 4 and Figure S7, HA and SA showed comparable inhibition effects, while BSA demonstrated much less inhibition on the adsorption of PFAS on PB(0.75). The difference can be caused by the different carboxylic acidities of BSA (1 mequiv/g), HA (3.4 mequiv/g), and SA (3.5 mequiv/g).^40^ The higher carboxylic acidities of HA and SA enable them to more effectively compete with PFAS for adsorption sites on PB(0.75) due to their greater density of negatively charged carboxyl groups. Similar trends were found in the removal of PFAS by nanofiltration in the presence of these three NOMs.^41^

A series of inorganic salts, 1 mM monovalent salt and 0.5 mM divalent salt, were also added to the solution to assess the influence of coexisting inorganic ions on the adsorption of PFAS by PB(0.75). Figure 4 shows the removal rate of PFAS after the introduction of different ions. Both cationic and anionic ions negatively affected the adsorption of PFAS. The introduction of inorganic salts increased the number of counterions in the solution, leading to the neutralization of the surface charge of PB(0.75) and hence weakening of the electrostatic interactions between PFAS and PB(0.75). Additionally, anions could compete with PFAS for adsorption sites, further hindering PFAS adsorption. When comparing the effects of monovalent and divalent ions, it becomes evident that divalent ions exhibit a much more significant inhibition effect on PFAS adsorption. Matrices containing CO_3_^2–^ and SO_4_^2–^ showed much stronger inhibition than those containing Cl^–^ and NO_3_^–^. Similarly, matrices with Na^+^, K^+^, and NH_4_^+^ demonstrated relatively weak adsorption inhibition compared to that of Mg^2^^+^ and Ca^2^^+^. The higher charge density of divalent ions enhances their ability to induce the aforementioned effects, resulting in a more pronounced inhibition of PFAS adsorption onto PB(0.75). Regarding the differences among various PFAS species, the trend is consistent with other matrix conditions: shorter-chain PFAS are more strongly impacted. This can be attributed to the dominant effect of electrostatic interactions on the removal of shorter-chain PFAS.

Taken together, these results indicate that PFAS uptake by PB(0.75) is governed primarily by electrostatic attraction—especially for short-chain species—while hydrophobic interactions become increasingly important with longer-chain lengths. Performance is maximized when electrostatic attraction between positively charged PB(0.75) and negatively charged PFAS is strongest, enabled by abundant adsorption sites, enhanced protonation under acidic conditions, and limited competition from other anionic species. In contrast, a high concentration of carboxyl-rich NOM (humics/alginate) and multivalent ions (Ca^2^^+^/Mg^2^^+^, SO_4_^2–^/CO_3_^2–^) significantly suppressed PFAS uptake on PB(0.75). On the bright side, conventional water treatment processes already target NOM removal (to reduce disinfection byproduct formation, taste/odor issues, and interference in downstream steps), and hardness mitigation (to reduce scale formation). Thus, PB(0.75) would be most effective when used downstream of such pretreatment steps, allowing it to focus on PFAS removal with fewer competing interferences.

### Modified Linear Solvation Energy Relationship (LSER) Model

The linear solvation energy relationship (LSER) is a pragmatic approach to understanding the partitioning process and has been successfully used to predict adsorption capacities toward a large array of compounds by carbon nanotubes,^42,43^ graphene oxide,^44^ and AC.^45,46^ As shown in Table 2, incorporating the ionization descriptor *P* in eqs 7 and 8 significantly enhanced the correlation coefficient and decreased the standard error of the estimates when compared to eqs 5 and 6, suggesting that *P* is essential in accurately describing the relationship. The positive coefficient of *P* also indicates that electrostatic attractions play an essential role in the adsorption between PFAS and PB(0.75), consistent with experimental observations. Between the two modified models, eq 7 slightly outperformed eq 8, as reflected by its higher *R* ^2^ and lower standard error of estimates.

**Table 2:** LSER Model Fitting Results^a^

eq	descriptor	adj. *R* ^2^	std error of the estimates	*c*	*e*	*s*	*a*	*b*	*v*	*l*	*p*
eq 5	E,S,A,B,V	0.595	0.4971	–1.255					1.905	N.A.	N.A.
eq 6	S,A,B,V,L	0.595	0.4971	–1.255	N.A.				1.905		N.A.
eq 7	E,S,A,B,V,P	0.988	0.0841	–2.923	2.805	–1.287			2. 981	N.A.	1.655
eq 8	S,A,B,V,L,P	0.941	0.1901	–5.387	N.A.	–0.457			1.754		3.268

a N.A.: not applicable, indicating that the corresponding descriptor does not apply to the equation. If a cell is blank, it means the descriptor is not considered significant.

All equations identified McGowan’s characteristic volume (*V*) as a positive contributor to the adsorption between PFAS and PB(0.75) in the aqueous phase, which agreed well with experimental observations and previous studies.^42–44^ The increase in molecular size with PFAS chain length leads to a higher energy requirement for cavity formation among water molecules, resulting in an enhanced hydrophobic repulsion from water onto carbon surfaces. Larger molecules also exhibit more pronounced time-dependent electron density fluctuations, which strengthen intermolecular forces such as van der Waals interactions. Consistent with this interpretation, eq 7 identifies *E*, a descriptor of dispersion interactions in polarizable compounds, as a positive contributor to the adsorption process, in agreement with the trend observed for longer-chain PFAS.^31^ By contrast, eq 8 did not retain *L*, another parameter correlated with van der Waals interactions, likely due to the high interrelationship between *V* and *L* (Figure S8).

Descriptors *A* and *B*, representing hydrogen-bond donor and acceptor capacities, were excluded from the model, suggesting that hydrogen bonding is not a dominant adsorption mechanism. Although F is the most electronegative element in the periodic table, the low polarization of the s and p electrons in F makes it a poor hydrogen-bond acceptor.^47^ The hydrogen-bonding capacity of PFAS mainly stems from the oxygen-containing functional groups and, therefore, remains constant for PFAS with the same functional group. S, which accounts for dipolarity/polarizability, decreased with the increase in the carbon chain length and was identified as a negative influence on the adsorption process. Overall, the insights obtained from LSER modeling align closely with experimental observations, providing a coherent mechanistic understanding of the adsorption of PFAS onto PB(0.75).

### SEM, FTIR, and XPS Analyses of PB(0.75) Before and After Adsorption

To probe the adsorption mechanism, PB(0.75) before and after PFAS adsorption was characterized and compared. The EDX elemental map shows strong N–F colocalization after PFOA adsorption (Figure 5a), implicating surface amines as adsorption sites, most likely via electrostatic attraction between anionic carboxylate/sulfonate headgroups and the cationic amines on PB(0.75). XPS survey spectra further confirm surface uptake as F is absent on pristine PB(0.75) and appears only after exposure (Figure 5b and Table S4). Notably, higher adsorption capacities corresponded to greater PFAS loading and, consequently, higher F content on the surface. For example, the Langmuir adsorption capacities of PB(0.75) for PFBS, PFHxS, and PFOS were 3, 454.5, 1213 mg/g, respectively, with corresponding F percentage of 8.77%, 9.85%, and 16.78%, demonstrating a positive correlation between F% and PFAS uptake. The deconvolution of the high-resolution XPS spectra (C 1s, N 1s, O 1s, and F 1s) is provided in Figures S9–S17 and summarized in Figure 5b. After adsorption, a new peak at 291.5 eV (C-4), which can be attributed to CF_3_ or highly fluorinated carbon environments,^48^ appeared in the high-resolution C 1s spectra of PB(0.75) materials except for those contacted with PFBA and GenX. The content percentage of C-4 is also correlated with adsorption capacity of PB(0.75) toward PFAS. Meanwhile, the percentage of N-2 and O-2, which is associated with (NH_2_) and (O–H), respectively, also increased after adsorption.

**Figure 5:**
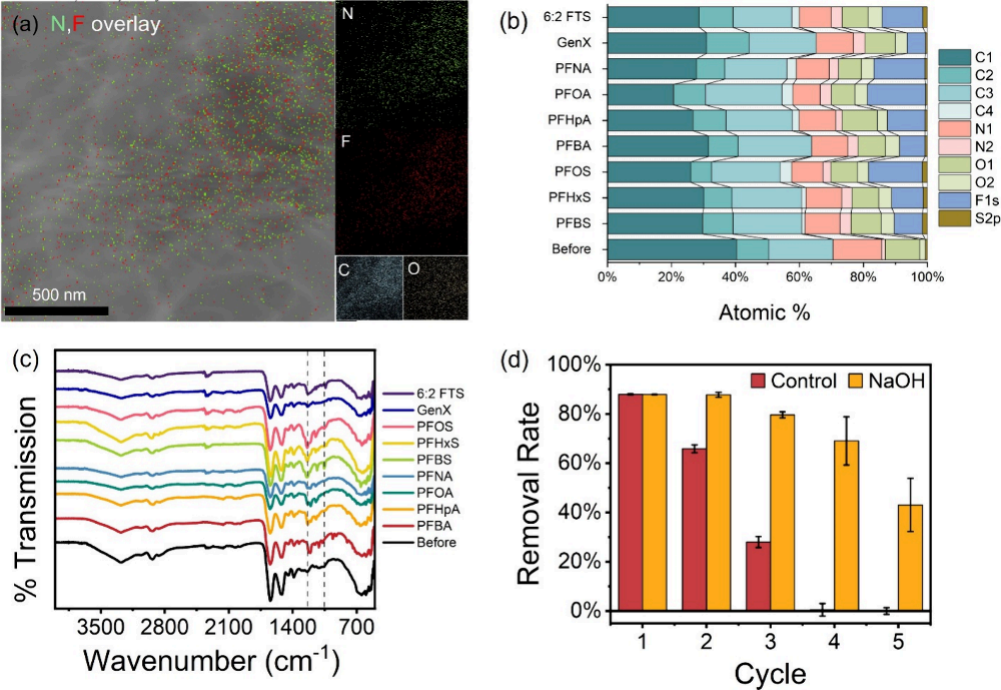
Characterization of PB(0.75) after adsorption. (a) EDX mapping after PFOA adsorption. (b) High-resolution XPS peak deconvolution results for PB(0.75) before and after PFAS exposure. (c) FTIR spectra of PB(0.75) before and after PFAS exposure. (d) Regeneration of PB(0.75) using alkaline washing.

FTIR spectroscopy was employed to investigate the functional groups of PB(0.75) after exposure to individual PFAS compounds. The characteristic bands of the protein backbone (O–H or N–H stretching (∼3292 cm^–1^), amide I (∼1643 cm^–1^), amide II (∼1516 cm^–1^), and amide III (∼1242 cm^–1^)) were preserved following PFAS exposure (Figure 5c), indicating that the protein structure remained intact. A closer examination of the 1300–900 cm^–1^ region revealed the emergence of several new peaks after adsorption (Figure S18). A distinct band at ∼1206 cm^–1^, corresponding to CF_2_/CF–C symmetric stretching, appeared in most PFAS-treated samples except for GenX and 6:2 FTS. Another PFAS-associated band emerged near 1150 cm^–1^, consistent with literature reports of CF_2_/CF_3_ stretching vibrations in perfluoroalkyl species. Additionally, a third band was observed around ∼1053 cm^–1^ in several sulfonated PFAS—PFBS (1055 cm^–1^), PFHxS (1029 cm^–1^), PFOS (1053 cm^–1^), and 6:2 FTS (1036 cm^–1^)—corresponding to the symmetric SO_3_^–^ stretching mode.^49^ These spectral features provide direct evidence of PFAS adsorption onto PB(0.75) without disrupting the underlying protein framework.

### Regeneration Results

Provided with the strong pH dependence of PFAS adsorption on PB(0.75) and the dominant role of electrostatic interactions, we hypothesize that the adsorption process should be reversible. In other words, by alteration of the pH, adsorbed PFAS could be released from the PB(0.75) surface, enabling subsequent adsorption cycles. Thus, a NaOH solution was used to regenerate the spent PB(0.75) in this study. To ensure the exhaustion of the adsorbent, 0.8 mg of PB(0.75) was mixed with 1.5 mL of PFOA solution (200 mg/L) for 1 h. The mixture was then centrifuged at 13,000 rpm to separate the adsorbent, which was subsequently washed with Milli-Q water until it reached a neutral pH. As shown in Figure 5d, the removal efficiency of PB(0.75) gradually decreased and reached complete exhaustion after three cycles without any regeneration measures. Meanwhile, NaOH successfully regenerated PB(0.75), restoring its adsorption efficiency to levels comparable to that of fresh adsorbent. In alkaline conditions, PB(0.75) becomes negatively charged, and PFAS were desorbed due to the electrostatic repulsion. At cycles 4 and 5, the regeneration efficiency declined, likely due to the mass loss of adsorbent during the extensive centrifugation processes, as evidenced by solids visible in the wash solution and a reduced pellet height after centrifugation. To mitigate mass loss, future work could explore strategies such as coupling the adsorbent with magnetic particles for magnetic separation, embedding it within membranes^9^ or other easily recoverable supports, or fabricating self-supporting monoliths,^50^ among others.

## Conclusions

Bovine serum albumin (BSA) was modified with poly­(ethylenimine) (PEI) via a facile cross-linking approach. The grafting of PEI significantly enhanced adsorption performance by imparting a positive surface charge to the surface of BSA. The optimal PEI:BSA mass ratio was determined to be 0.75, and the resulting PB(0.75) was evaluated for the removal of PFBA, PFHpA, PFOA, PFNA, PFBS, PFHxS, PFOS, GenX, and 6:2 FTS. Notably, the Langmuir maximum adsorption capacities are greater than 1000 mg/g for PFOA (1111), PFOS (1213), PFNA (1330), and PFHpA (1215), ranking among the highest reported in the literature. In addition, PB(0.75) showed markedly faster adsorption kinetics, with pseudo-second-order rate constants exceeding 5 g/(mg·min) for PFOA (11.2), PFNA (15.7), PFHxS (11.6), and 6:2 FTS (5.4), far surpassing those of activated carbons. For PFBA, PFBS, and GenX, the improvements were more modest but still evident, highlighting the need for future optimization to further enhance the adsorption of these emerging PFAS. Adsorption studies under various initial PFAS concentrations, pH levels, natural organic matter types and concentrations, and different inorganic ions showed that PB(0.75) performs well for long-chain PFAS under a wide range of conditions. Analysis of the adsorption data using kinetic and isotherm models revealed that the adsorption process occurs on a heterogeneous surface and is primarily driven by the remaining adsorption capacity. The relationship between the adsorption capacity and the interactions between PFAS and PB(0.75) was successfully elucidated by a modified linear solvation energy relationship (LSER) model with an additional ionization descriptor. Electrostatic interactions were found to be the primary mechanism for PFAS removal, while hydrophobic interactions also contribute significantly, especially for more hydrophobic PFAS. XPS, FTIR, and EDS mapping of PB(0.75) after adsorption highlighted the pivotal contribution of positively charged amine groups introduced by PEI. Additionally, the ability to reversibly desorb PFAS under alkaline conditions allows for multiple reuses of the spent adsorbent, reducing both PFAS-laden hazardous waste generation and operational costs. Taken together, the high adsorption efficiency, tolerance to diverse water chemistries, and efficient generation of PB(0.75) point to significant potential for real-world PFAS remediation. More broadly, PEI-functionalized proteins may be engineered to target a wider range of anionic micropollutants beyond PFAS by tuning the amine density, tailoring cross-linking architecture, and selecting appropriate protein backbones.

## Supplementary Material



## Data Availability

The data supporting the findings of this study are available within the paper and its Supporting Information. All other data are available from the authors upon request.

## Notes

Introduction to the LSER model, additional adsorbent characterization results, analytic details, and time-evolution data for those adsorption experiments whose end point values are used to construct the manuscript heatmap (PDF)
