# Supplementary material for: Rapid, High-Capacity, and Reusable Bovine Serum Albumin-Based Adsorbents for Perfluoroalkyl and Polyfluoroalkyl Substance Removal
Source: ACS Appl Mater Interfaces. 2026 Jan 16;18(3):5026–37. doi: 10.1021/acsami.5c13467 (PMC12862774; doi:10.1021/acsami.5c13467)
Supplement: Supplementary file 1 [file am5c13467_si_001.pdf]

**Supporting Information**

# Rapid, High-Capacity, and Reusable Bovine Serum Albumin-Based Adsorbent for PFAS Removal

Liqing Yan, Elliot Reid, Zefang Chen, Jiahao He, Yongsheng Chen*

School of Civil and Environmental Engineering, Georgia Institute of Technology, 200 Bobby Dodd Way, Atlanta, GA, 30332, United States

* Corresponding author: Yongsheng Chen, Email: yongsheng.chen@ce.gatech.edu

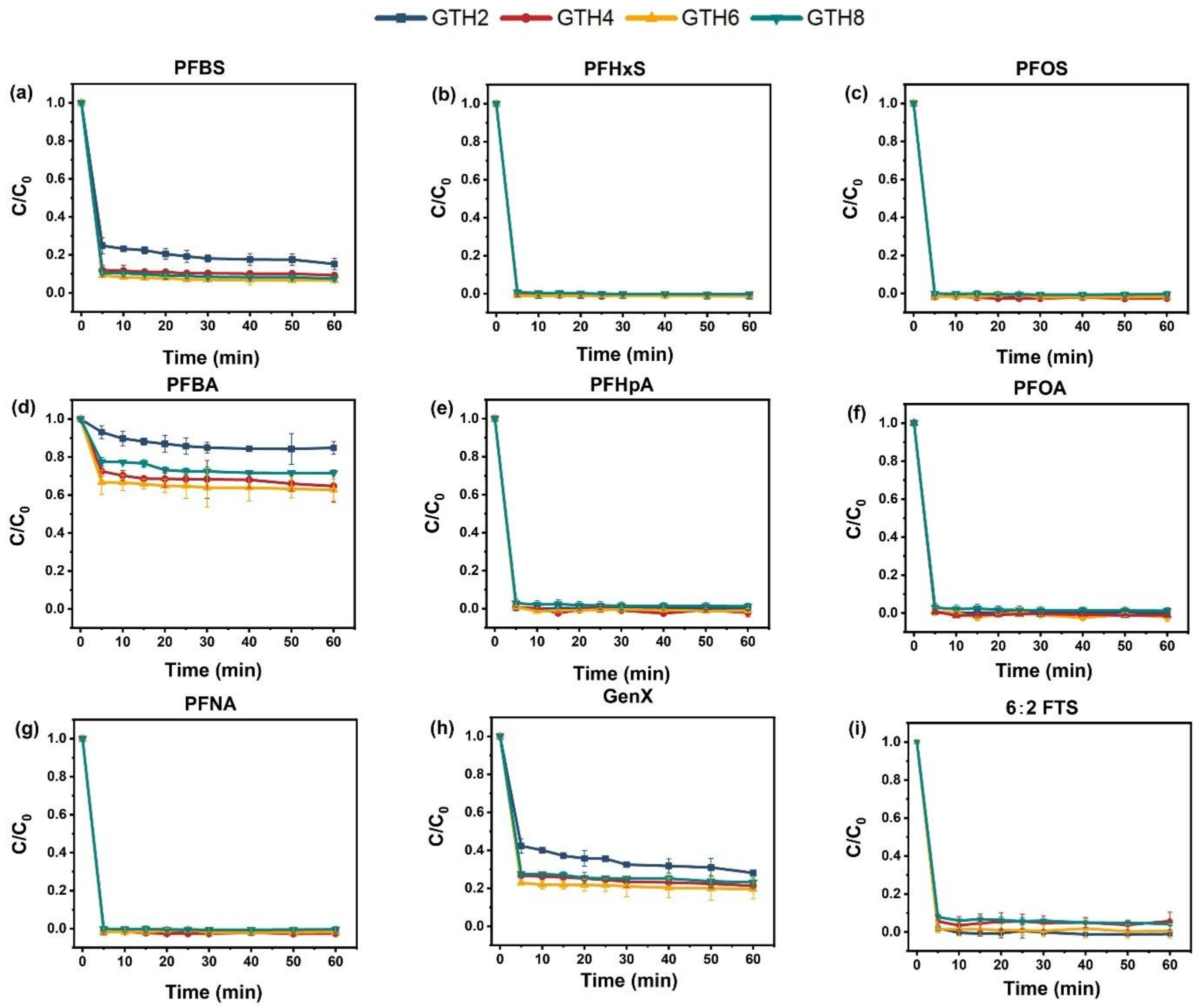


**Fig. S1** The influences of different GTH:BSA mass ratio on the removal rate of (a) PFBS (b) PFHxS, (c) PFOS, (d) PFBA, (e) PFHpA, (f) PFOA, (g) PFNA, (h) GenX, (i) 6:2 FTS on PB(0.75). PEI:BSA mass ratio = 0.75, PFAS initial concentration: 100 μg/L, adsorbent dosage: 200 mg/L.

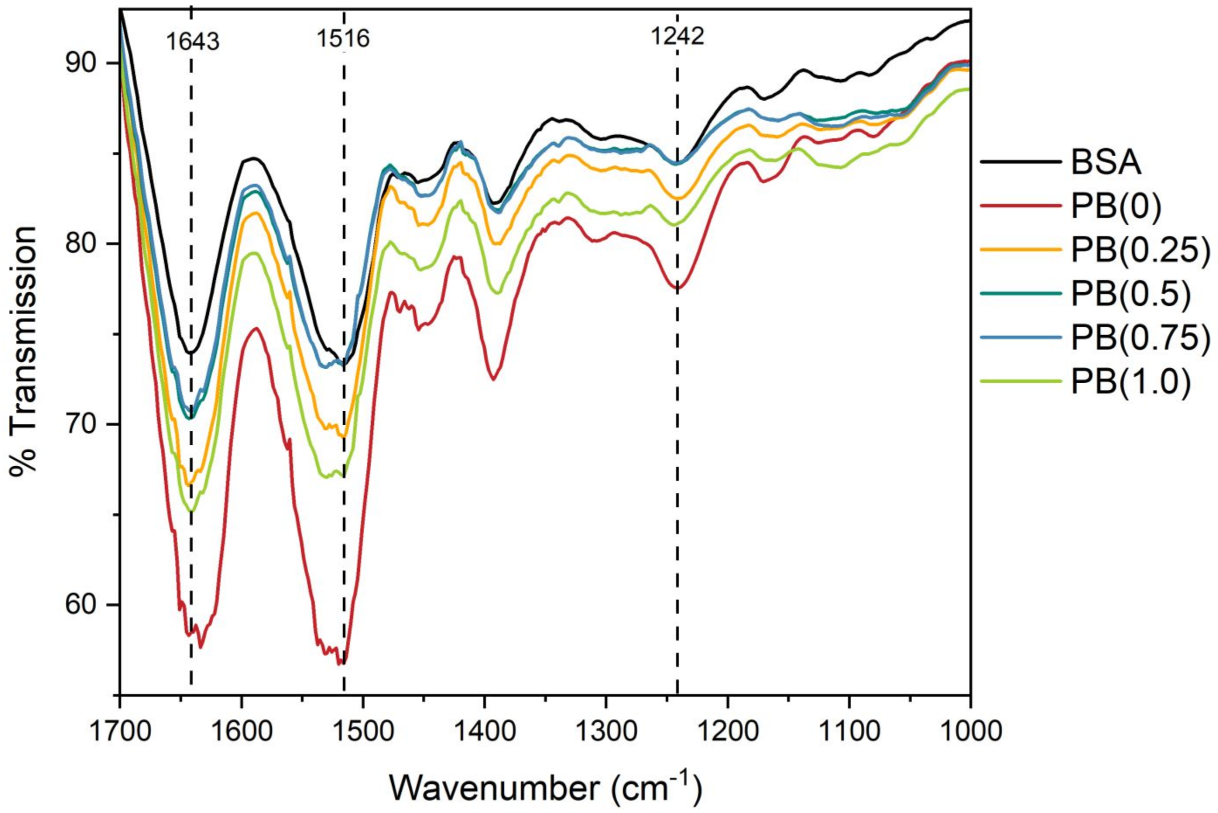


**Fig. S2** Enlarged FTIR spectra of prepared PB(x) around 1700-1000 $cm^{-1}$.

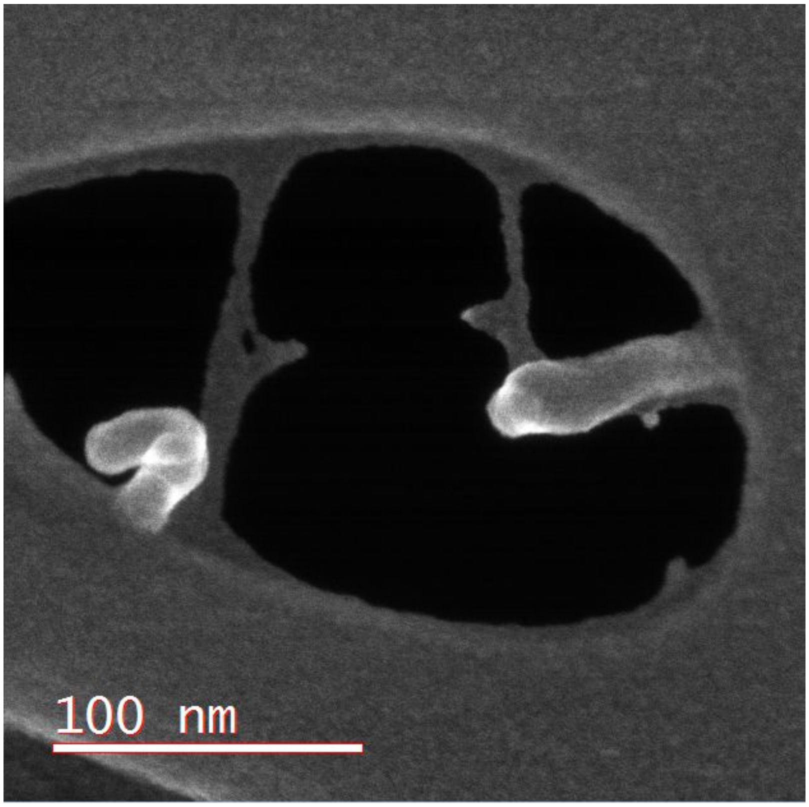


**Fig. S3** SEM image of PB(0.75) with low sample concentration (0.5 mg/mL).

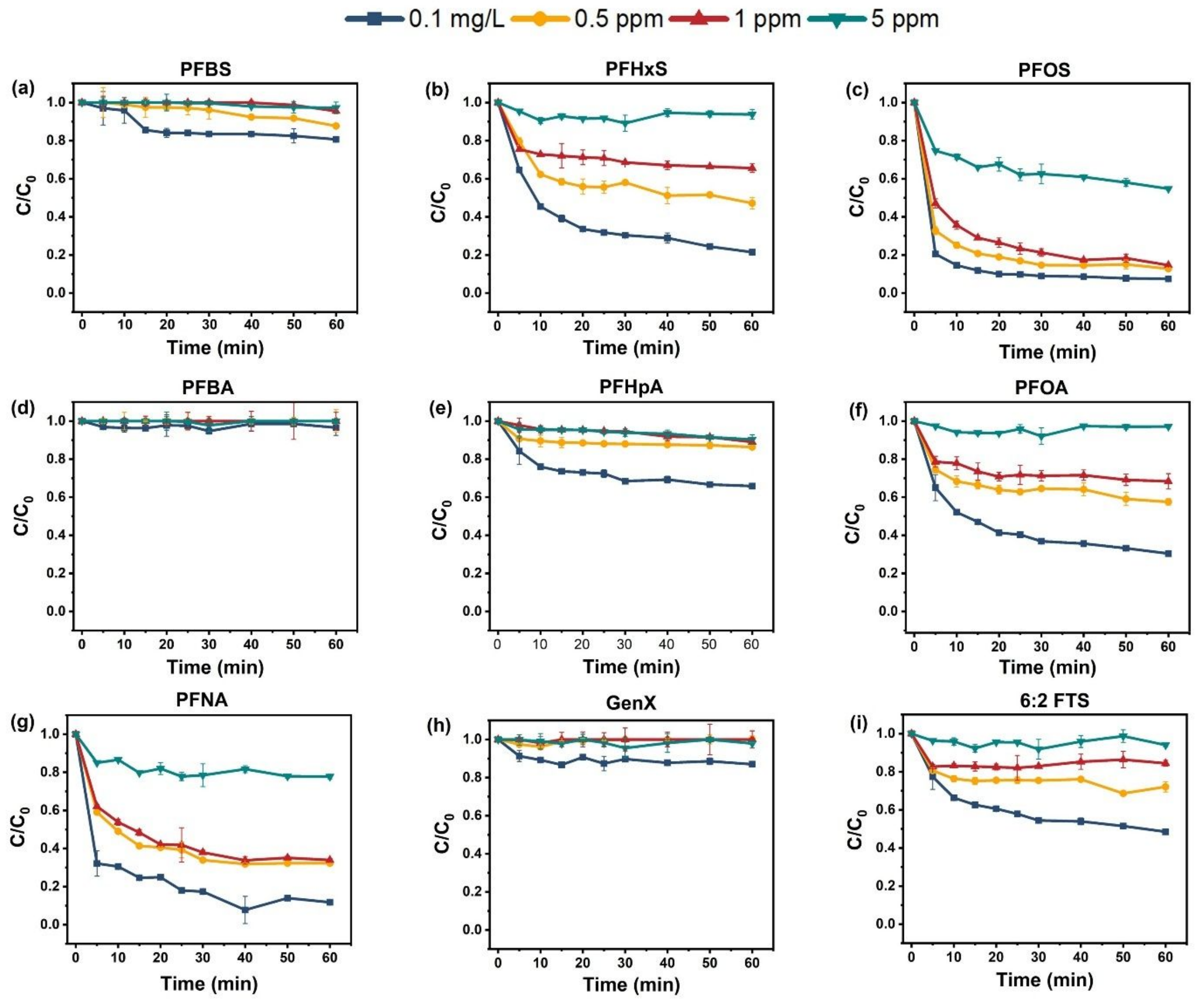


**Fig. S4** The influence of PFAS initial concentration on the removal rate of (a) PFBS (b) PFHxS, (c) PFOS, (d) PFBA, (e) PFHpA, (f) PFOA, (g) PFNA, (h) GenX, and (i) 6:2 FTS on PB(0.75).

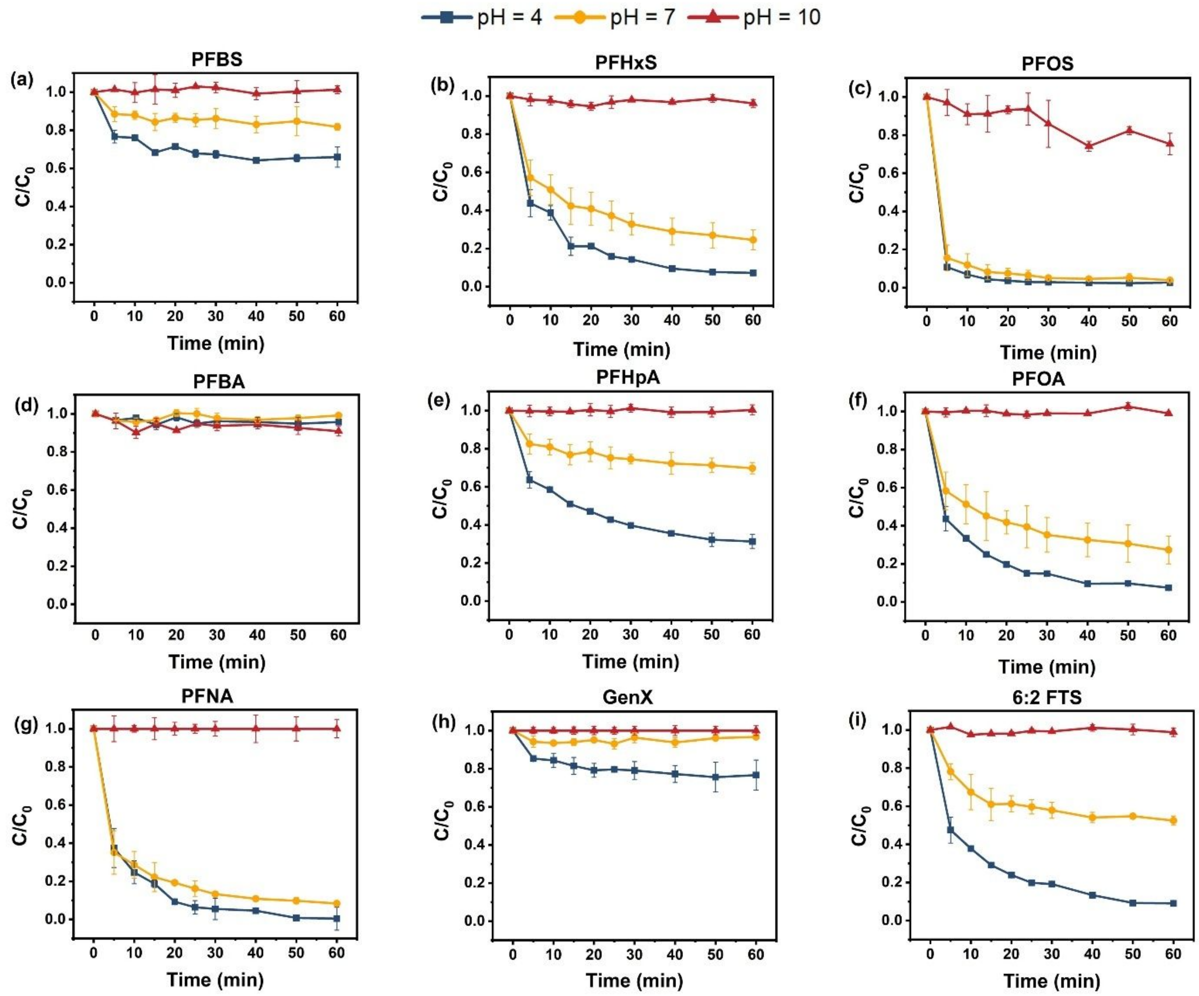


**Fig. S5** The influences of pH on the removal rate of (a) PFBS (b) PFHxS, (c) PFOS, (d) PFBA, (e) PFHpA, (f) PFOA, (g) PFNA, (h) GenX, and (i) 6:2 FTS on PB(0.75).

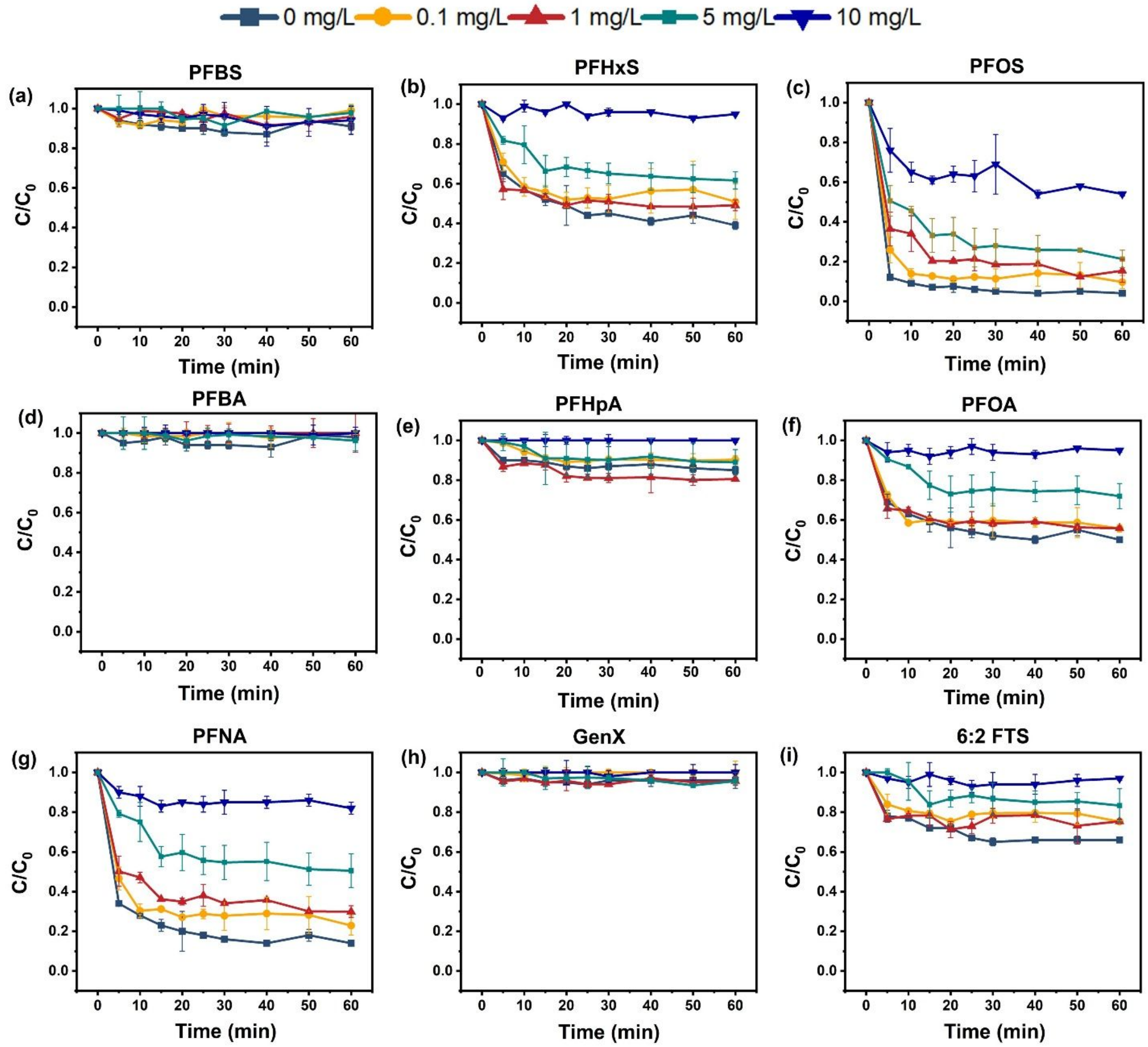


**Fig. S6** The influences of HA concentration on the removal rate of (a) PFBS (b) PFHxS, (c) PFOS, (d) PFBA, (e) PFHpA, (f) PFOA, (g) PFNA, (h) GenX, and (i) 6:2 FTS on PB(0.75).

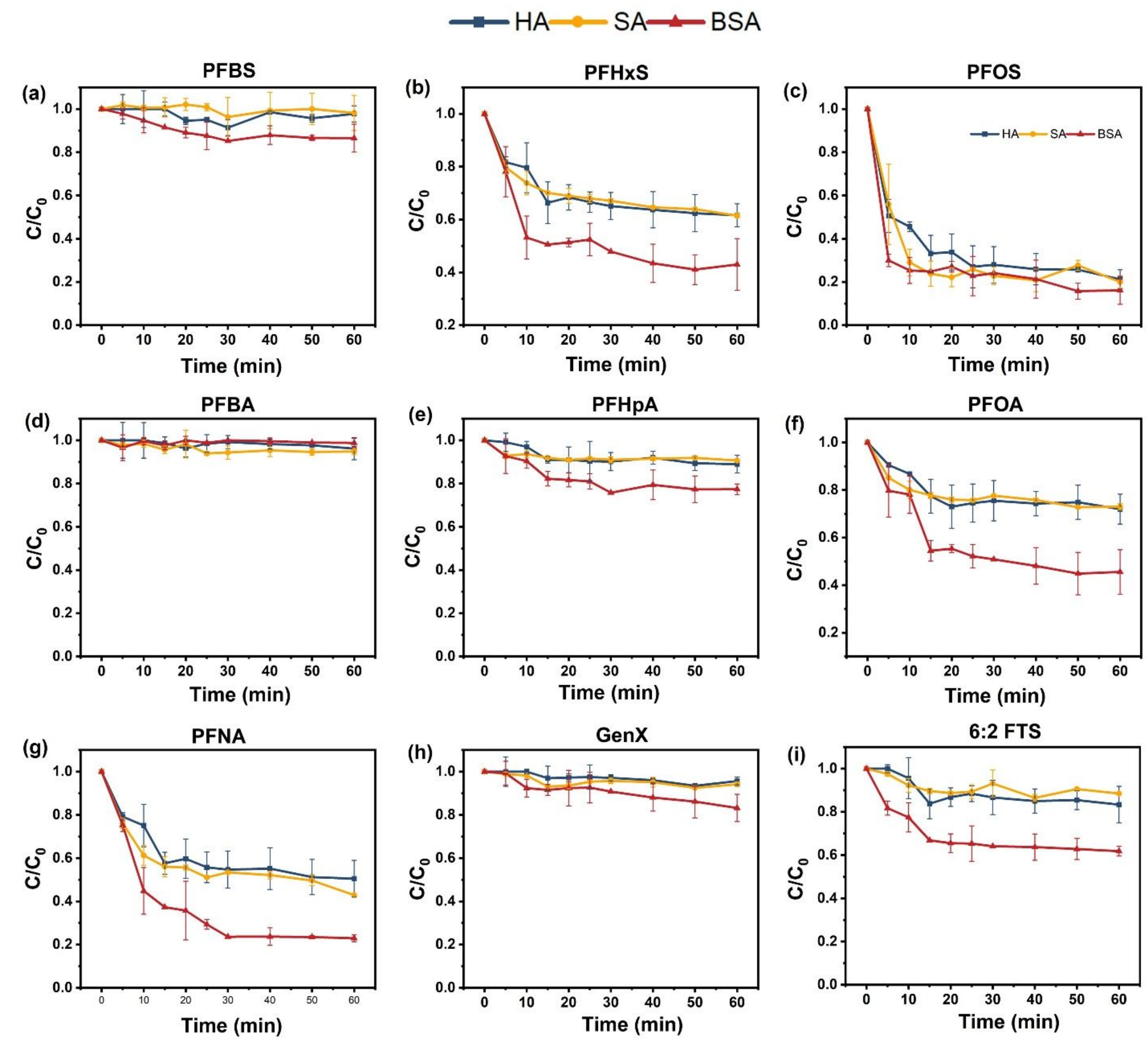


**Fig. S7** The influences of NOM type on the removal rate of (a) PFBS (b) PFHxS, (c) PFOS, (d) PFBA, (e) PFHpA, (f) PFOA, (g) PFNA, (h) GenX, and (i) 6:2 FTS on PB(0.75).

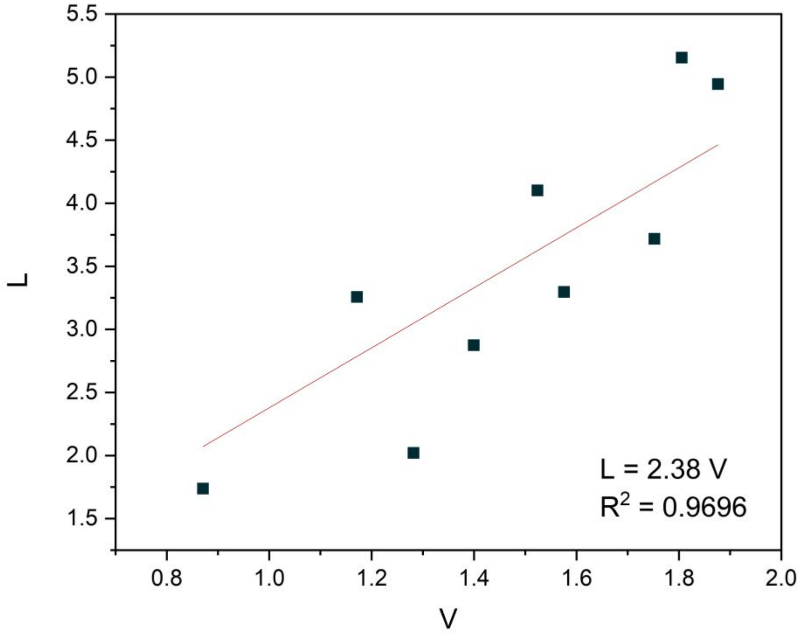


**Fig. S8** Intercorrelation between V and L descriptors.

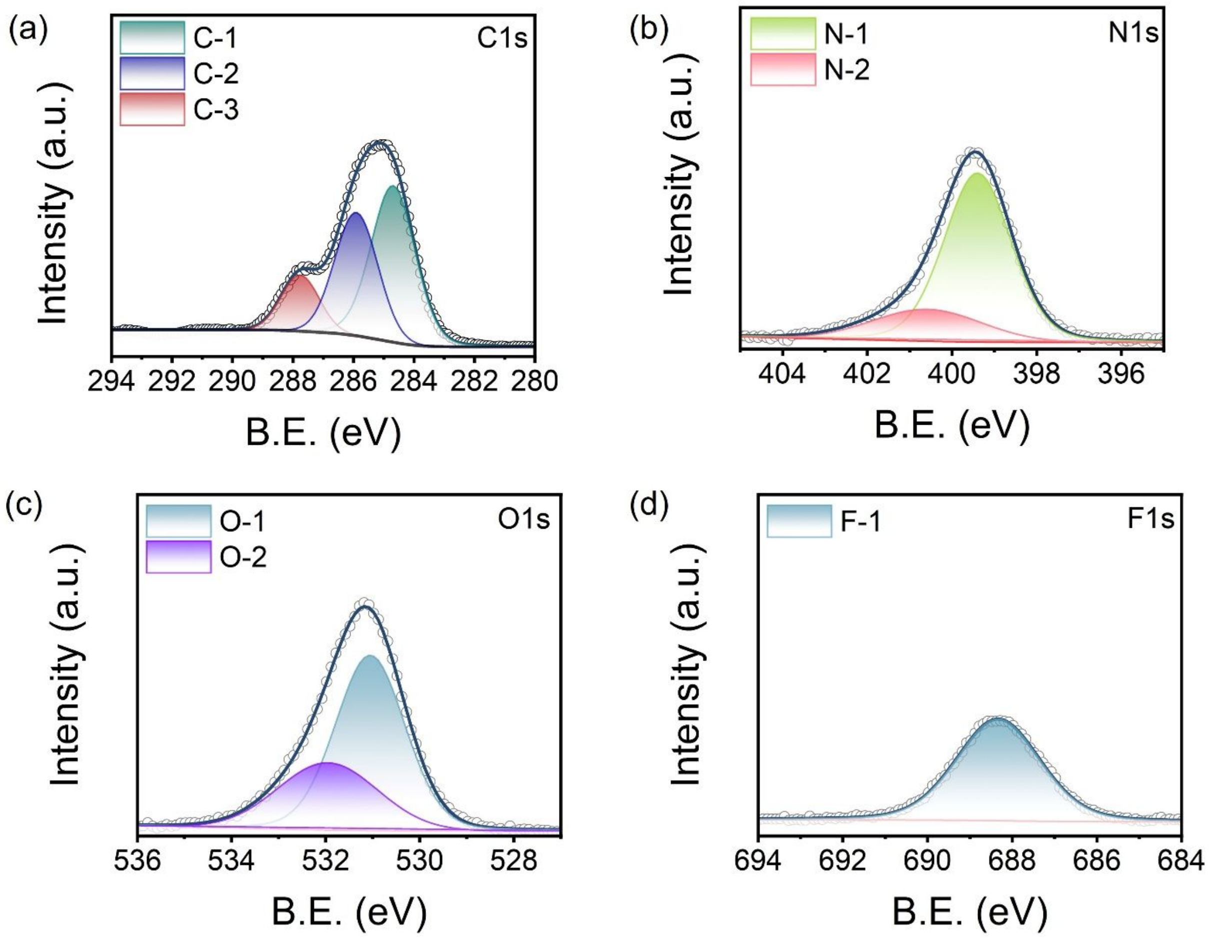


**Fig. S9** High-resolution XPS spectra of PB(0.75) after PFBA adsorption: (a) C 1s, (b) N 1s, (c) O 1s, (d) F 1s.

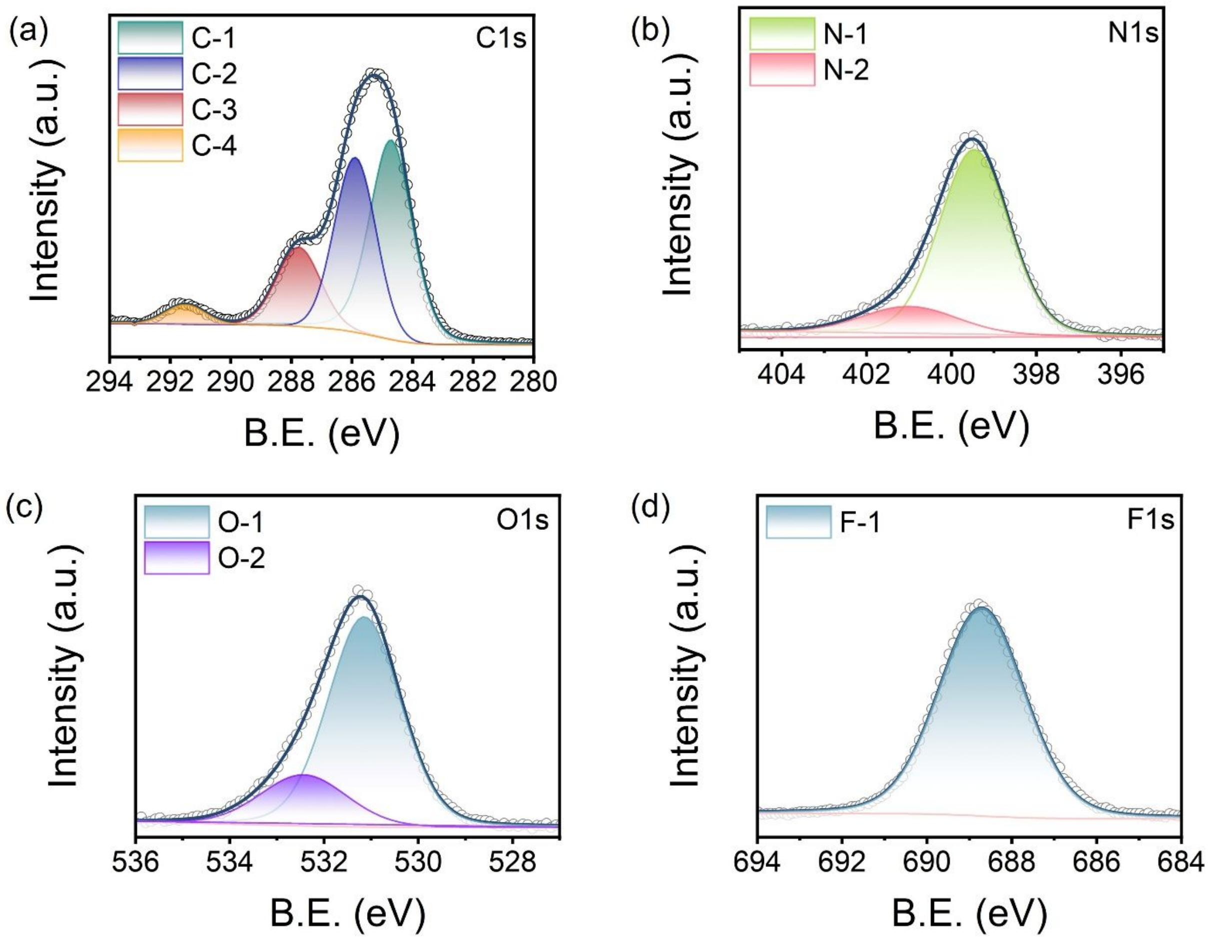


**Fig. S1** High resolution XPS spectra of PB(0.75) after adsorption of PFHpA: (a) C 1s, (b) N 1s, (c) O 1s, (d) F 1s.

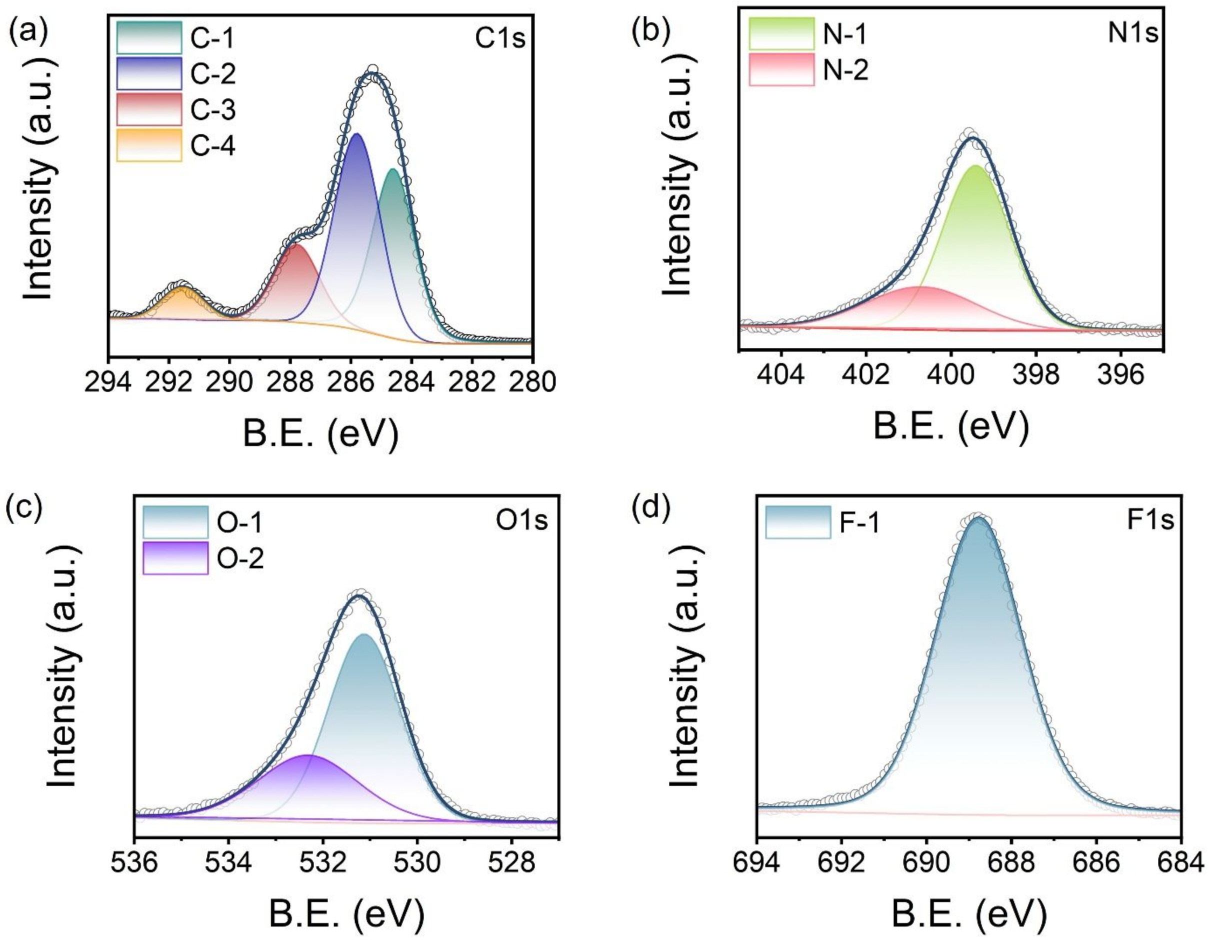


**Fig. S2** High resolution XPS spectra of PB(0.75) after adsorption of PFOA: (a) C 1s, (b) N 1s, (c) O 1s, (d) F 1s.

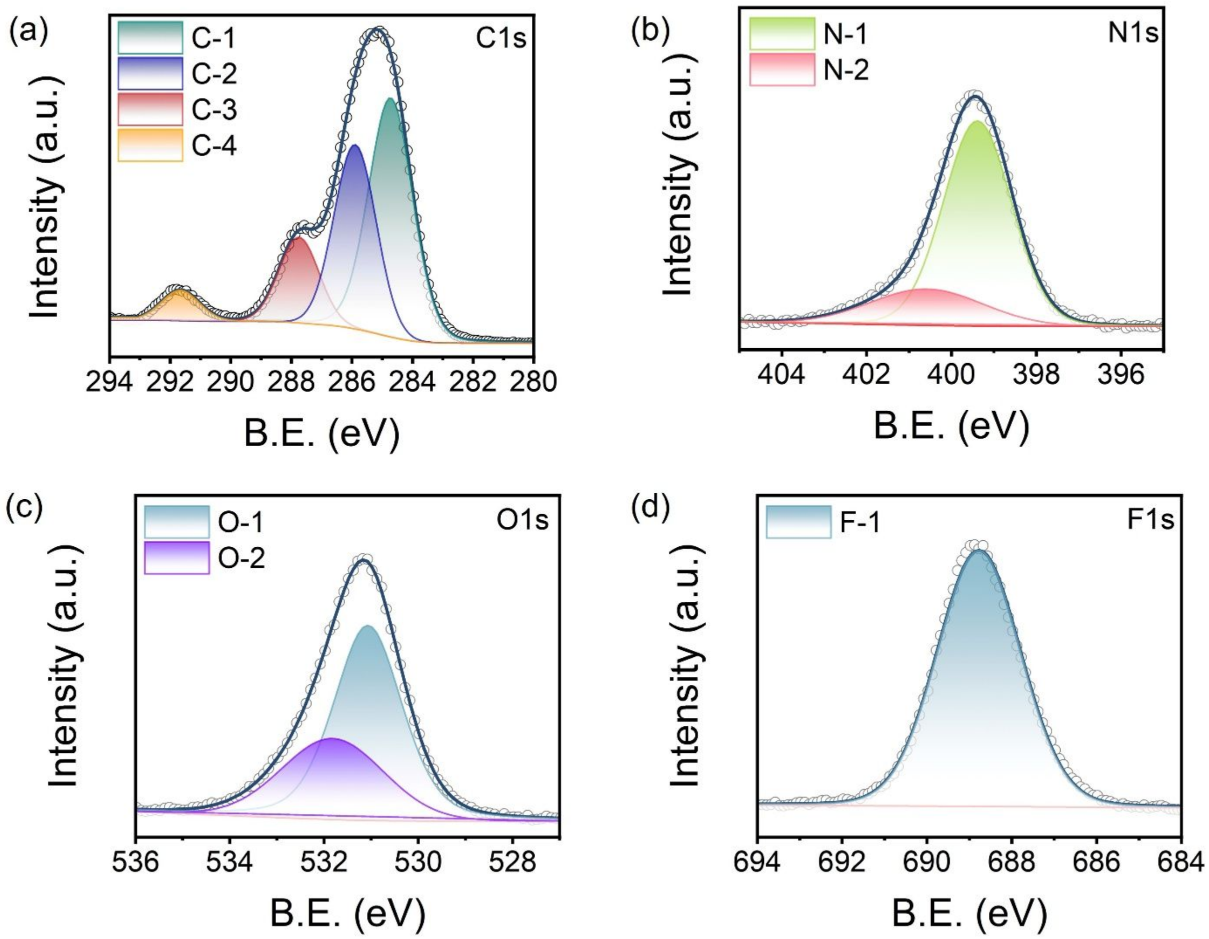


**Fig. S32** High resolution XPS spectra of PB(0.75) after adsorption of PFNA: (a) C 1s, (b) N 1s, (c) O 1s, (d) F 1s.

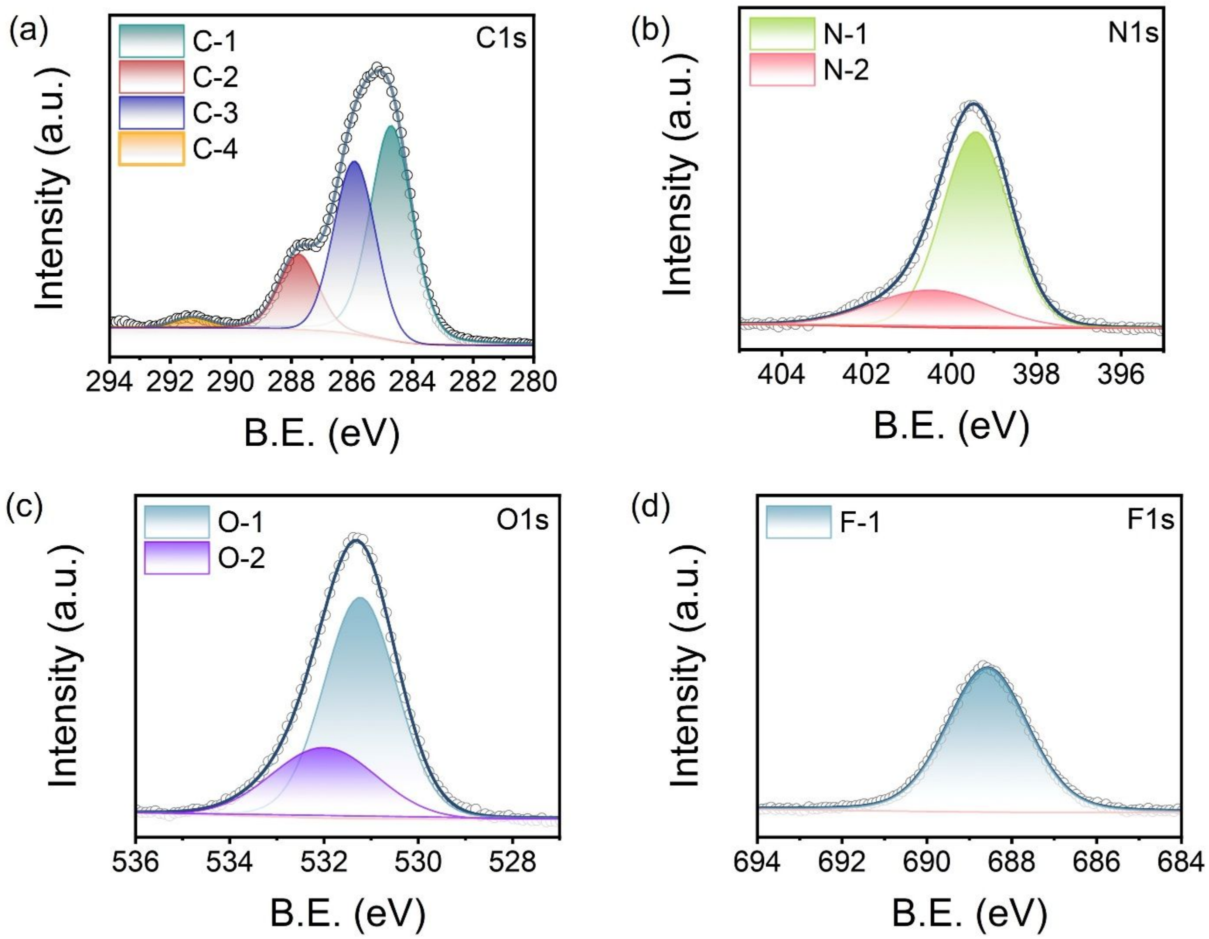


**Fig. S43** High resolution XPS spectra of PB(0.75) after adsorption of PFBS: (a) C 1s, (b) N 1s, (c) O 1s, (d) F 1s.

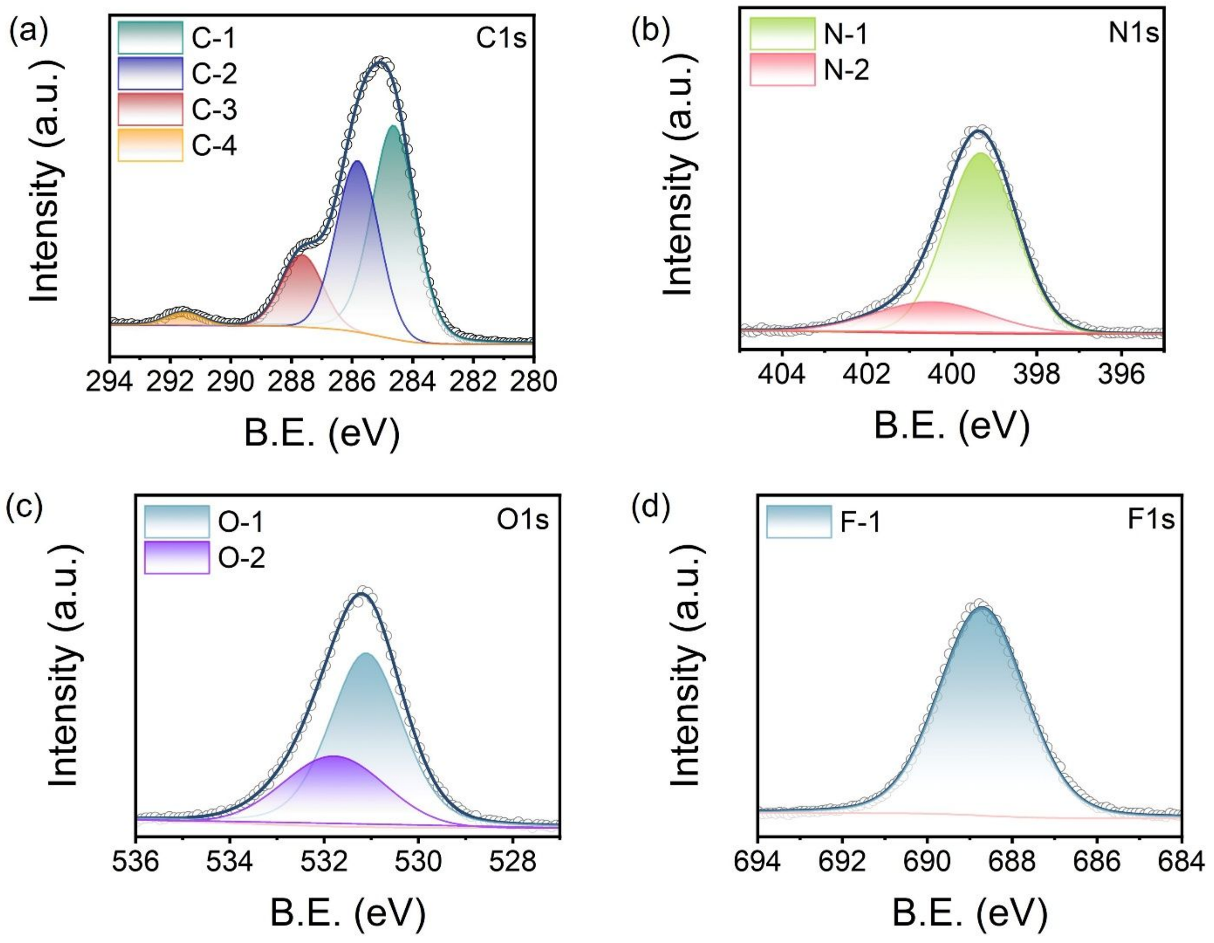


**Fig. S54** High resolution XPS spectra of PB(0.75) after adsorption of PFHxS: (a) C 1s, (b) N 1s, (c) O 1s, (d) F 1s.

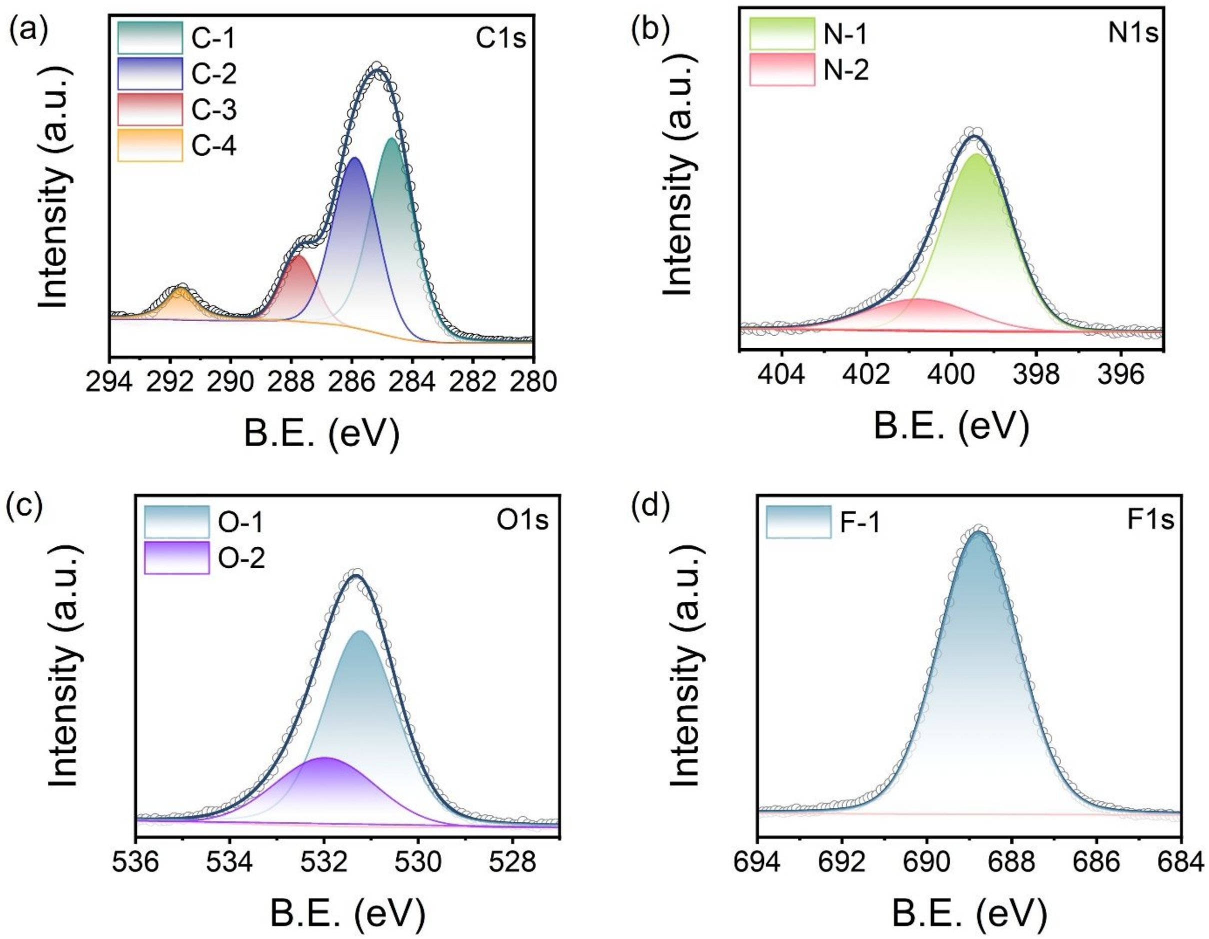


**Fig. S65** High resolution XPS spectra of PB(0.75) after adsorption of PFOS: (a) C 1s, (b) N 1s, (c) O 1s, (d) F 1s.

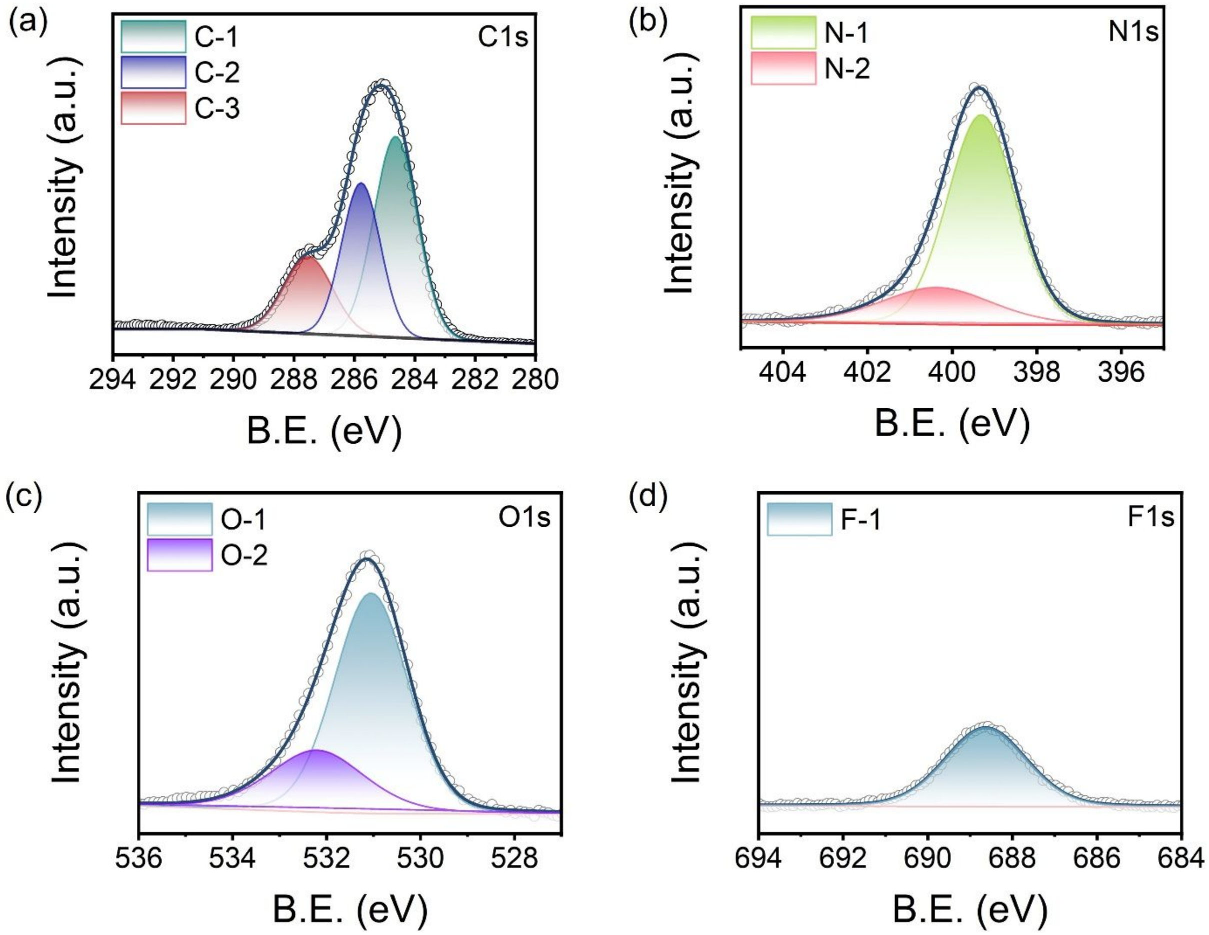


**Fig. S76** High resolution XPS spectra of PB(0.75) after adsorption of GenX: (a) C 1s, (b) N 1s, (c) O 1s, (d) F 1s.

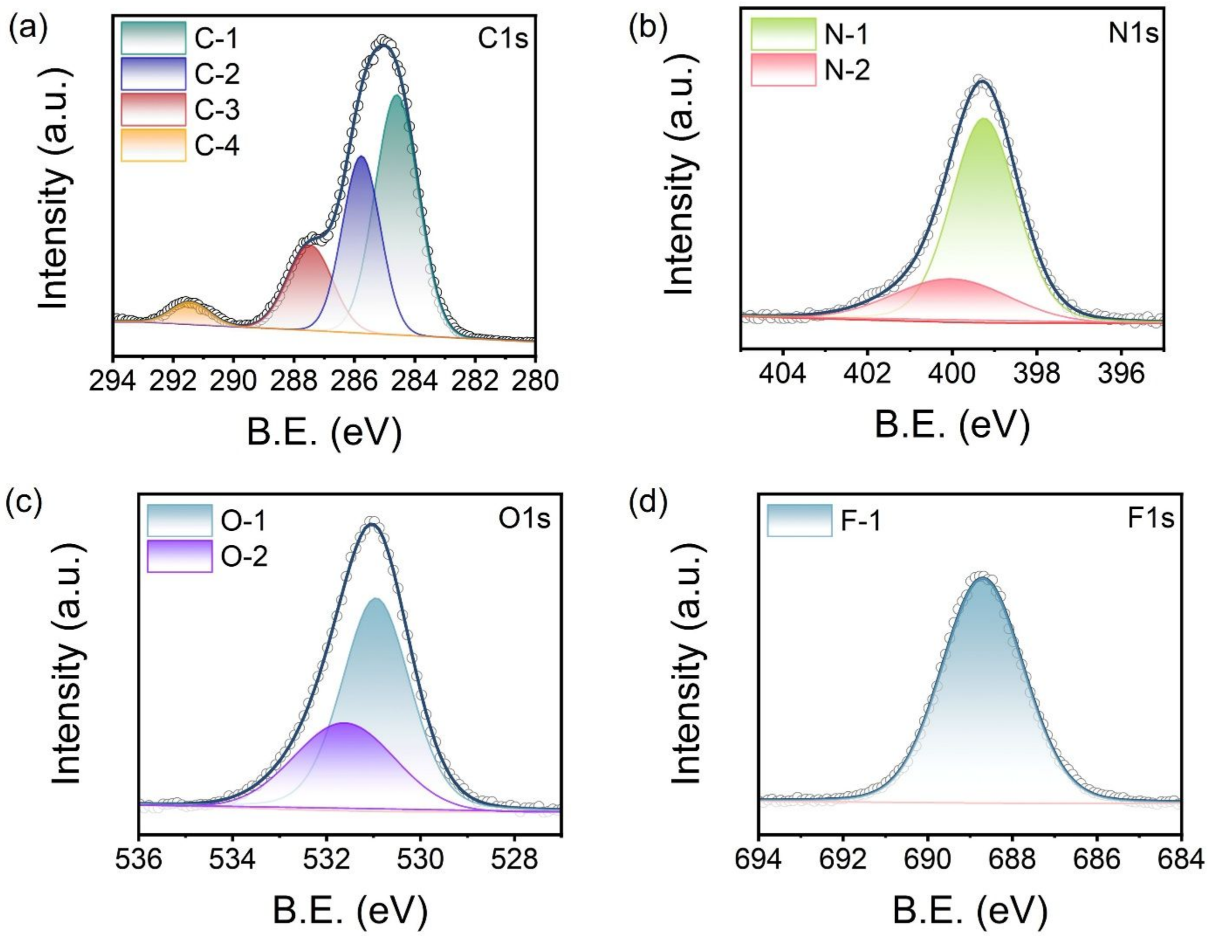


**Fig. S87** High resolution XPS spectra of PB(0.75) after adsorption of 6:2 FTS: (a) C 1s, (b) N 1s, (c) O 1s, (d) F 1s.

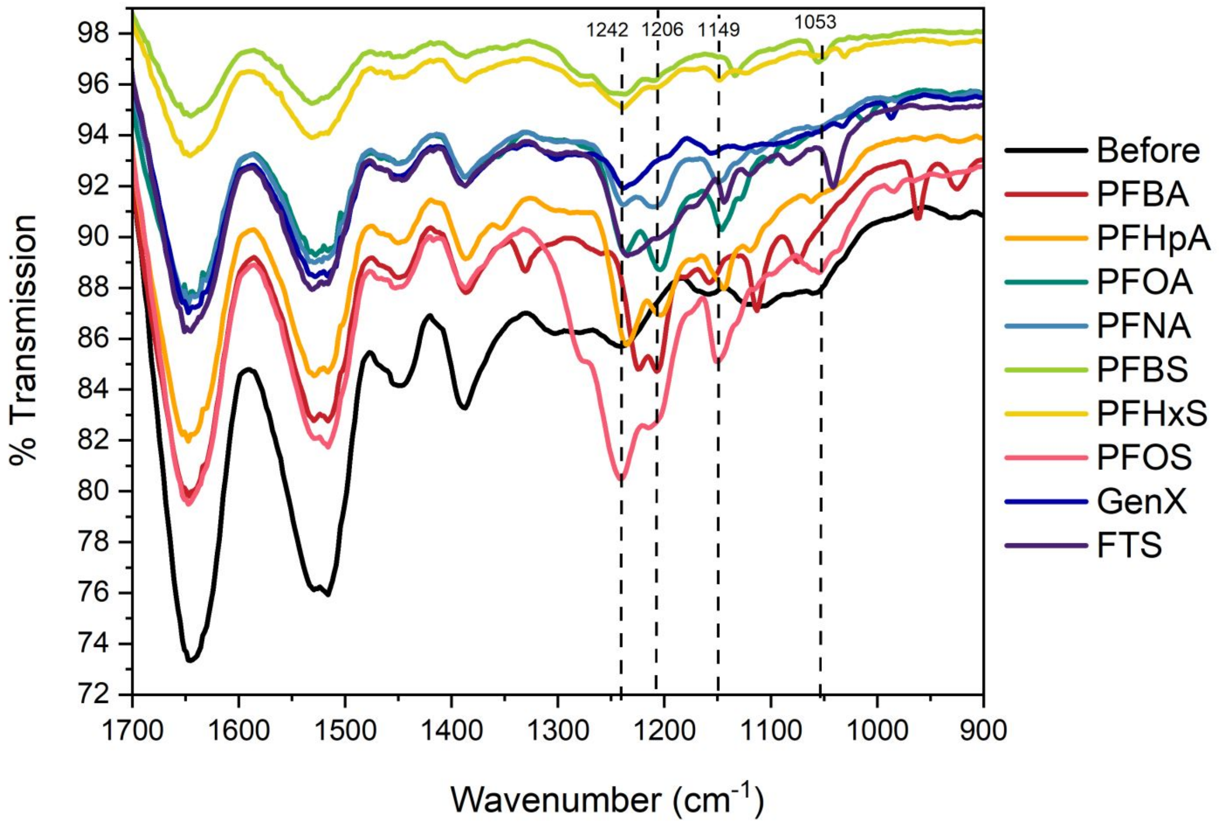


**Fig. S18** Enlarged FTIR spectra of PB(0.75) before and after adsorbed with different PFAS around 1700 - 900 $cm^{-1}$.

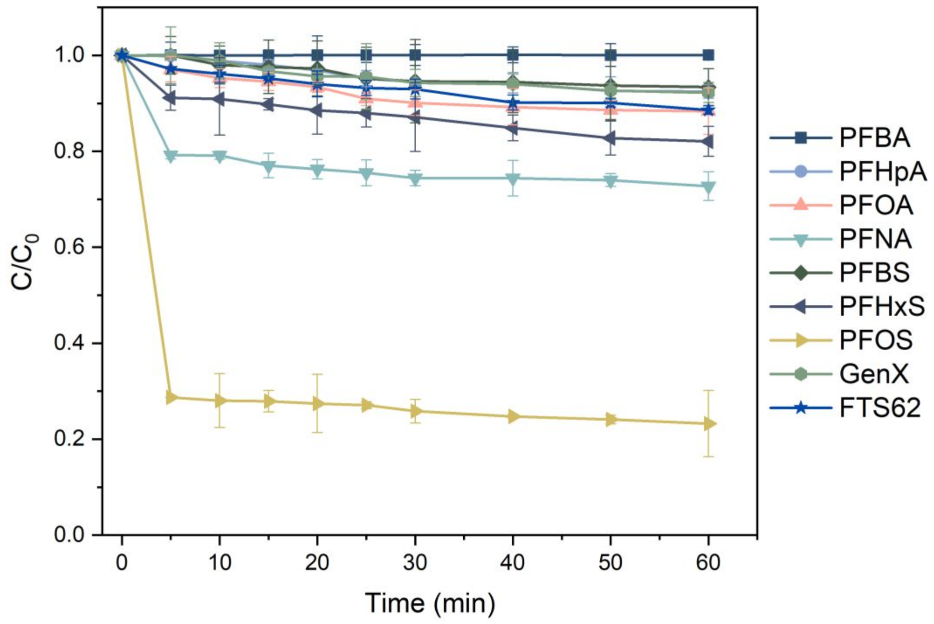


**Fig. S19** Time course of PFAS concentration in adsorbent-free controls after filtration through a PES filter.

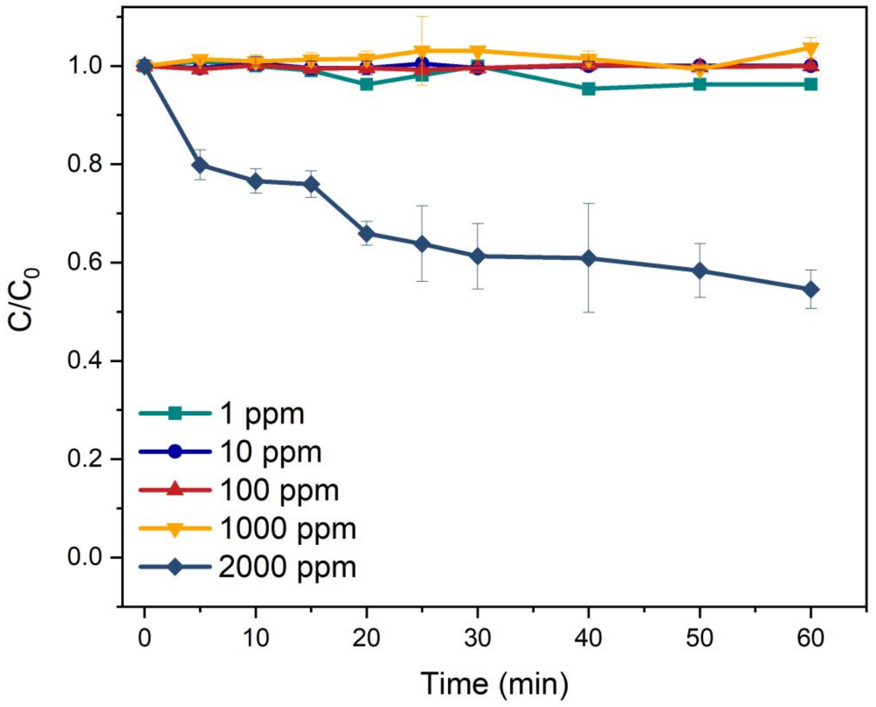


**Fig. S20** Time course of PFNA concentration in adsorbent-free controls after centrifugation.

Table S1 PFASs investigated in this work.

| No | Category | Analyte name | Abbr. | Formula | Chain length | Structure |
|---|---|---|---|---|---|---|
| 1 | Perfluorinated alkyl carboxylic acids (PFCAs) | Perfluorobutanoic acid | PFBA | $C_4HF_7O_2$ | 4 | 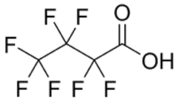 |
| 2 | | Perfluoroheptanoic acid | PFHpA | $C_7HF_{13}O_2$ | 7 | 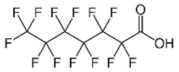 |
| 3 | | Perfluorooctanoic acid | PFOA | $C_8HF_{15}O_2$ | 8 | 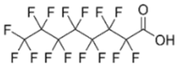 |
| 4 | | Perfluorononanoic acid | PFNA | CF3(CF2)7COOH | 9 | 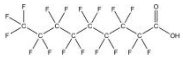 |
| 5 | Perfluorinated alkyl sulfonic acids (PFSAs) | Perfluorobutanesulfonic acid | PFBS | $C_4HF_9O_3S$ | 4 | 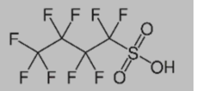 |
| 6 | | Perfluorohexanesulfonic acid | PFHxS | $C_6HF_{13}O_3S$ | 6 | 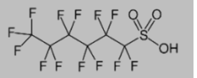 |
| 7 | | Perfluorooctanesulfonic acid | PFOS | $C_8HF_{17}O_3S$ | 8 | 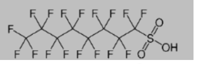 |
| 8 | Replacement PFAS | Ammonium salt of hexafluoropropylene oxide dimer acid | GenX | $C_6HF_{11}O_3$ | 6 | 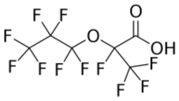 |
| 9 | | 6:2 Fluorotelomer sulfonic acid | 6:2 FTS | $C_8H_5F_{13}O_3S$ | 8 | 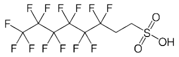 |

Table S2 Kinetic fitting results

| | $\text{In}(q_e - q_t) = \text{In}q_e - k_1 t$ | | | $\frac{t}{q_t} = \frac{1}{k_2 q_e^2} + \frac{t}{q_e}$ | | |
|---|---|---|---|---|---|---|
| | $k_1$ ($min^{-1}$) | $q_e$ (mg/g) | $R^2$ | $k_2$ (g/(mg.min)) | $q_e$ (mg/g) | $R^2$ |
| PFBA | 0.038 | 0.12 | 0.9471 | 0.554 | 0.24 | 0.9897 |
| PFHpA | 0.042 | 0.02 | 0.9731 | 4.672 | 0.51 | 0.9999 |
| PFOA | 0.052 | 0.01 | 0.9874 | 11.195 | 0.46 | 1.0000 |
| PFNA | 0.061 | 0.01 | 0.7899 | 15.654 | 0.39 | 1.0000 |
| PFBS | 0.039 | 0.03 | 0.9485 | 3.349 | 0.49 | 0.9999 |
| PFHxS | 0.092 | 0.01 | 0.9513 | 11.584 | 0.45 | 1.0000 |
| PFOS | 0.066 | 0.07 | 0.9654 | 2.148 | 0.46 | 1.0000 |
| GenX | 0.023 | 0.06 | 0.9662 | 1.457 | 0.45 | 0.9982 |
| 6:2 FTS | 0.074 | 0.03 | 0.9529 | 5.350 | 0.53 | 1.0000 |

Table S3 Compare Freundlich isotherm parameters with other adsorbents.

| PFAS | Adsorbent | $q_e = K_F C_e^{1/n}$ | | | Ref. |
|---|---|---|---|---|---|
| | | $K_F$ | 1/n | $R^2$ | |
| PFBA | PB(0.75) | 3.10 | 0.99 | 0.9946 | This work |
| | All silica zeolite β | 0.098 | 1.0 | 0.986 | [4] |
| | reed straw-derived biochar | 2.05 | 0.35 | 0.98 | 5 |
| | GAC | 0.34 | 0.59 | 0.97 | 5 |
| | Hydrotalcite | 1.35 | 1.22 | 0.99 | 6 |
| PFHpA | PB(0.75) | 78.61 | 0.60 | 0.9793 | This work |
| | Coconut based activated carbon | 33.12 | 0.3 | 0.99 | 7 |
| | Bituminous coal based activated carbon | 53.26 | 0.32 | 0.94 | 7 |
| | GAC | 6.79 | 0.89 | 0.99 | 8 |
| | GAC | 19.77 | 0.383 | 0.9354 | 9 |
| PFOA | PB(0.75) | 197.20 | 0.40 | 0.9778 | This work |
| | PEI-c-CMC | 11.46 | 0.51 | 0.99 | 10 |
| | Crosslinked β-cyclodextrin | 68 | 0.25 | 0.99 | 11 |
| | All silica zeolite β | 97 | 1.4 | 0.958 | [4] |
| | Fluorinated-Squaramide COF | 19.34 | 0.512 | 0.933 | 12 |
| | Ni8-Pyrazolate Porous Framework | 70.177 | 0.296 | 0.939 | 13 |
| | reed straw-derived biochar | 8.41 | 0.27 | 0.95 | 5 |
| | GAC | 4.09 | 0.44 | 0.95 | 5 |
| | Coconut based activated carbon | 23.04 | 0.33 | 0.97 | 7 |
| | Bituminous coal based activated carbon | 112.82 | 0.28 | 0.92 | 7 |
| | Hydrotalcite | 62.85 | 1.19 | 0.96 | 6 |
| | GAC | 29.05 | 0.115 | 0.6142 | 9 |
| PFNA | PB(0.75) | 216.77 | 0.46 | 0.9598 | This work |
| | Hydrotalcite | 149.21 | 1.2 | 0.99 | 6 |
| | GAC | 18.11 | 0.52 | 0.9954 | 9 |
| PFBS | PB(0.75) | 5.28 | 0.83 | 0.9901 | This work |
| | All silica zeolite β | 2.4 | 1.1 | 0.994 | [4] |
| | Reed straw-derived biochar | 6.02 | 0.22 | 0.96 | 5 |
| | GAC | 0.66 | 0.49 | 0.98 | 5 |
| | Coconut based activated carbon | 0.35 | 0.54 | 0.92 | 7 |
| | Bituminous coal based activated carbon | 7.66 | 0.41 | 0.92 | 7 |
| | Hydrotalcite | 0.03 | 1.56 | 0.99 | 6 |

Table S3 Continued

| PFAS | Adsorbent | $q_e = K_F C_e^{1/n}$ | | | Ref. |
|---|---|---|---|---|---|
| | | $K_F$ | 1/n | $R^2$ | |
| PFHxS | PB(0.75) | 77.27 | 0.41 | 0.9834 | This work |
| | Reed straw-derived biochar | 7.37 | 0.21 | 0.92 | 5 |
| | GAC | 1.73 | 0.48 | 0.9 | 5 |
| | Coconut based activated carbon | 3.47 | 0.45 | 0.98 | 7 |
| | Bituminous coal based activated carbon | 19.55 | 0.4 | 0.95 | 7 |
| | Hydrotalcite | 10.88 | 1.4 | 0.99 | 6 |
| | GAC | 33.20 | 0.164 | 0.7481 | 9 |
| PFOS | PB(0.75) | 230.71 | 0.48 | 0.9140 | This work |
| | Willow-derived chars | 5.23 | 0.492 | 0.992 | 14 |
| | Maize straw chars | 7.27 | 0.459 | 0.986 | 14 |
| | Maize straw ash | 26.8 | 0.571 | 0.951 | 14 |
| | SWCNT | 122 | 0.324 | 0.998 | 14 |
| | MWCNT | 47.1 | 0.437 | 0.991 | 14 |
| | All silica zeolite β | 74 | 1.3 | 0.900 | 4 |
| | Fluorinated-Squaramide COF | 31.61 | 0.448 | 0.942 | 12 |
| | Amine-functionalized olefin-linked COF | 5.885 | 0.606 | 0.9920 | 15 |
| | Ni8-Pyrazolate Porous Framework | 35.862 | 0.307 | 0.924 | 13 |
| | Reed straw-derived biochar | 11.43 | 0.3 | 0.92 | 5 |
| | GAC | 5.55 | 0.51 | 0.93 | 5 |
| | Bamboo-Derived Biochar – vacuum pyrolysis | 131.0 | 0.32 | 0.998 | 16 |
| | Bamboo-Derived Biochar –pyrolysis | 11.8 | 0.67 | 0.974 | 16 |
| | Coconut based activated carbon | 101.3 | 0.23 | 0.94 | 7 |
| | Bituminous coal based activated carbon | 142.73 | 0.27 | 0.86 | 7 |
| | Hydrotalcite | 6.27 | 1.17 | 0.95 | 6 |
| | GAC | 26.79 | 0.315 | 0.9286 | 9 |
| GenX | PB(0.75) | 3.65 | 0.87 | 0.9921 | This work |
| | Crosslinked β-cyclodextrin | 321 | 0.09 | 0.96 | 11 |
| | Fluorinated-Squaramide COF | 12.33 | 0.578 | 0.938 | 12 |
| | Amine-Functionalized COF | 64 | 0.28 | 0.97 | 17 |
| | Treated activated carbon | 2.88 | 1.30 | 0.988 | 18 |
| 6:2 FTS | PB(0.75) | 25.95 | 0.92 | 0.9903 | This work |
| | Bamboo-Derived Biochar –pyrolysis | 50 | 0.19 | 0.948 | 16 |
| | Hydrotalcite | 76.51 | 1.06 | 0.88 | 6 |
| | Colloidal activated carbon | 3.9 | 0.2 | - | 19 |

Note: KF unit: (mg/g)/(mg/L)$^{1/n}$, COF: covalent organic framework.

Table S4 XPS survey scan results of PB(0.75) before and after adsorption

| | N1s | C1s | O1s | F1s | S2p |
|---|---|---|---|---|---|
| Before | 16.32 | 70.51 | 12.54 | 0 | 0.64 |
| PFBA | 14.47 | 63.85 | 12.99 | 7.97 | 0.72 |
| PFHpA | 13.49 | 59.93 | 14.14 | 11.76 | 0.68 |
| PFOA | 12.03 | 57.86 | 11.22 | 18.16 | 0.55 |
| PFNA | 13.2 | 59.06 | 11.01 | 16 | 0.74 |
| PFBS | 14.39 | 61.73 | 13.52 | 8.77 | 1.58 |
| PFHxS | 14.39 | 61.99 | 12.5 | 9.85 | 1.27 |
| PFOS | 12.36 | 57.54 | 11.71 | 16.78 | 1.61 |
| GenX | 15.2 | 64.87 | 13.05 | 5.7 | 0.61 |
| 6:2 FTS | 13.41 | 59.97 | 12.48 | 12.72 | 1.42 |

Table S5 mobile phase gradient for LC-MS/MS

| Time (min) | A% | B% |
|---|---|---|
| 0 | 90 | 10 |
| 2 | 90 | 10 |
| 4 | 30 | 70 |
| 16 | 2 | 98 |
| 18.5 | 100 | 0 |
| 23.5 | 100 | 0 |

Table S6 Descriptors used in the present study

| PFAS | pKa[20] | E | S | A | B | V | L | P | logKF |
|---|---|---|---|---|---|---|---|---|---|
| PFBA | 0.40 | -0.47 | 0.10 | 0.46 | 0.33 | 0.87 | 1.74 | 1.36 | 0.49 |
| PFHpA | -0.15 | -0.70 | -0.27 | 0.46 | 0.33 | 1.40 | 2.88 | 1.42 | 1.90 |
| PFOA | -0.20 | -0.88 | -0.39 | 0.46 | 0.33 | 1.58 | 3.30 | 1.42 | 2.29 |
| PFNA | -0.21 | -1.03 | -0.51 | 0.46 | 0.33 | 1.75 | 3.72 | 1.42 | 2.34 |
| PFBS | 0.14 | -0.42 | 0.74 | 0.31 | 0.75 | 1.17 | 3.26 | 1.39 | 0.72 |
| PFHxS | 0.14 | -0.50 | 0.49 | 0.31 | 0.75 | 1.52 | 4.10 | 1.39 | 1.89 |
| PFOS | 0.14 | -0.83 | 0.25 | 0.31 | 0.75 | 1.88 | 4.95 | 1.39 | 2.36 |
| GenX | 2.80 | -0.78 | 0.00 | 0.46 | 0.53 | 1.28 | 2.02 | 1.12 | 0.56 |
| 6:2 FTS | 1.31 | -0.69 | 0.94 | 0.31 | 0.75 | 1.81 | 5.16 | 1.27 | 1.41 |

## Reference


(1) Poole, C. F.; Atapattu, S. N. Recent Advances for Estimating Environmental Properties for Small Molecules from Chromatographic Measurements and the Solvation Parameter Model. *J. Chromatogr. A* **2023**, *1687*, 463682. https://doi.org/10.1016/j.chroma.2022.463682.

(2) Goss, K.-U. Predicting the Equilibrium Partitioning of Organic Compounds Using Just One Linear Solvation Energy Relationship (LSER). *Fluid Phase Equilibria* **2005**, *233* (1), 19–22. https://doi.org/10.1016/j.fluid.2005.04.006.

(3) Endo, S. Intermolecular Interactions, Solute Descriptors, and Partition Properties of Neutral Per- and Polyfluoroalkyl Substances (PFAS). *Environ. Sci. Technol.* **2023**, *57* (45), 17534–17541. https://doi.org/10.1021/acs.est.3c07503.

(4) Lauwers, A.; Vercammen, J.; De Vos, D. Adsorption of PFAS by All-Silica Zeolite β: Insights into the Effect of the Water Matrix, Regeneration of the Material, and Continuous PFAS Adsorption. *ACS Appl. Mater. Interfaces* **2023**, *15* (45), 52612–52621. https://doi.org/10.1021/acsami.3c12321.

(5) Liu, N.; Wu, C.; Lyu, G.; Li, M. Efficient Adsorptive Removal of Short-Chain Perfluoroalkyl Acids Using Reed Straw-Derived Biochar (RESCA). *Sci. Total Environ.* **2021**, *798*, 149191. https://doi.org/10.1016/j.scitotenv.2021.149191.

(6) Kim, H.-H.; Koster van Groos, P. G.; Zhao, Y.; Pham, A. L.-T. Removal of PFAS by Hydrotalcite: Adsorption Mechanisms, Effect of Adsorbent Aging, and Thermal Regeneration. *Water Res.* **2024**, *260*, 121925. https://doi.org/10.1016/j.watres.2024.121925.

(7) Cantoni, B.; Turolla, A.; Wellmitz, J.; Ruhl, A. S.; Antonelli, M. Perfluoroalkyl Substances (PFAS) Adsorption in Drinking Water by Granular Activated Carbon: Influence of Activated Carbon and PFAS Characteristics. *Sci. Total Environ.* **2021**, *795*, 148821. https://doi.org/10.1016/j.scitotenv.2021.148821.

(8) Zhang, D.; Luo, Q.; Gao, B.; Chiang, S.-Y. D.; Woodward, D.; Huang, Q. Sorption of Perfluorooctanoic Acid, Perfluorooctane Sulfonate and Perfluoroheptanoic Acid on Granular Activated Carbon. *Chemosphere* **2016**, *144*, 2336–2342. https://doi.org/10.1016/j.chemosphere.2015.10.124.

(9) Choi, H.; Jeon, J. Chemical Decomposition Combined with Physical Adsorption for the Treatment of Investigation-Derived Waste Containing PFAS.

(10) Ateia, M.; Attia, M. F.; Maroli, A.; Tharayil, N.; Alexis, F.; Whitehead, D. C.; Karanfil, T. Rapid Removal of Poly- and Perfluorinated Alkyl Substances by Poly(Ethylenimine)-Functionalized Cellulose Microcrystals at Environmentally Relevant Conditions. *Environ. Sci. Technol. Lett.* **2018**, *5* (12), 764–769. https://doi.org/10.1021/acs.estlett.8b00556.

(11) Yang, A.; Ching, C.; Easler, M.; Helbling, D. E.; Dichtel, W. R. Cyclodextrin Polymers with Nitrogen-Containing Tripodal Crosslinkers for Efficient PFAS Adsorption. *ACS Mater. Lett.* **2020**, *2* (9), 1240–1245. https://doi.org/10.1021/acsmaterialslett.0c00240.

(12) Huang, J.; Shi, Y.; Huang, G.; Huang, S.; Zheng, J.; Xu, J.; Zhu, F.; Ouyang, G. Facile Synthesis of a Fluorinated-Squaramide Covalent Organic Framework for the Highly Efficient and Broad-Spectrum Removal of Per- and Polyfluoroalkyl Pollutants. *Angew. Chem. Int. Ed.* **2022**, *61* (31), e202206749. https://doi.org/10.1002/anie.202206749.

(13) Li, K.; Hu, J.; Gu, Q.; He, J.; Peng, Y.-K.; Xu, Z. Removing Perfluoro Pollutants PFOA and PFOS by Two-Pronged Design of a Ni8-Pyrazolate Porous Framework. *ACS Appl. Mater. Interfaces* **2023**, *15* (29), 35107–35116. https://doi.org/10.1021/acsami.3c07568.

(14) Chen, X.; Xia, X.; Wang, X.; Qiao, J.; Chen, H. A Comparative Study on Sorption of Perfluorooctane Sulfonate (PFOS) by Chars, Ash and Carbon Nanotubes. *Chemosphere* **2011**, *83* (10), 1313–1319. https://doi.org/10.1016/j.chemosphere.2011.04.018.
(15) Song, X.; Wang, R.; Wang, X.; Han, H.; Qiao, Z.; Sun, X.; Ji, W. An Amine-Functionalized Olefin-Linked Covalent Organic Framework Used for the Solid-Phase Microextraction of Legacy and Emerging per- and Polyfluoroalkyl Substances in Fish. *J. Hazard. Mater.* **2022**, *423*, 127226. https://doi.org/10.1016/j.jhazmat.2021.127226.
(16) Ao, W.; Mian, M. M.; Zhang, Q.; Zhou, Z.; Deng, S. Bamboo-Derived Low-Cost Mesoporous Biochar for Efficient Removal of Per- and Polyfluoroalkyl Substances from Contaminated Water. *ACS EST Water* **2024**, *4* (6), 2711–2720. https://doi.org/10.1021/acsestwater.4c00211.
(17) Ji, W.; Xiao, L.; Ling, Y.; Ching, C.; Matsumoto, M.; Bisbey, R. P.; Helbling, D. E.; Dichtel, W. R. Removal of GenX and Perfluorinated Alkyl Substances from Water by Amine-Functionalized Covalent Organic Frameworks. *J. Am. Chem. Soc.* **2018**, *140* (40), 12677–12681. https://doi.org/10.1021/jacs.8b06958.
(18) Zhu, Y.; Ji, H.; He, K.; Blaney, L.; Xu, T.; Zhao, D. Photocatalytic Degradation of GenX in Water Using a New Adsorptive Photocatalyst. *Water Res.* **2022**, *220*, 118650. https://doi.org/10.1016/j.watres.2022.118650.
(19) Carey, G. R.; Hakimabadi, S. G.; Singh, M.; McGregor, R.; Woodfield, C.; Van Geel, P. J.; Pham, A. L.-T. Longevity of Colloidal Activated Carbon for in Situ PFAS Remediation at AFFF-Contaminated Airport Sites. *Remediat. J.* **2022**, *33* (1), 3–23. https://doi.org/10.1002/rem.21741.
(20) Daglar, H.; Keskin, S. Recent Advances, Opportunities, and Challenges in High-Throughput Computational Screening of MOFs for Gas Separations. *Coord. Chem. Rev.* **2020**, *422*, 213470. https://doi.org/10.1016/j.ccr.2020.213470.
